# Induction of the Unfolded Protein Response Drives Enhanced Metabolism and Chemoresistance in Glioma Cells

**DOI:** 10.1371/journal.pone.0073267

**Published:** 2013-08-15

**Authors:** Laura M. Epple, Rebecca D. Dodd, Andrea L. Merz, Anjelika M. Dechkovskaia, Matthew Herring, Benjamin A. Winston, Alex M. Lencioni, Rae L. Russell, Helen Madsen, Meheret Nega, Nathaniel L. Dusto, Jason White, Darell D. Bigner, Christopher V. Nicchitta, Natalie J. Serkova, Michael W. Graner

**Affiliations:** 1 Department of Neurosurgery, Anschutz Medical Center, University of Colorado Denver, Aurora, Colorado, United States of America; 2 Cell and Molecular Biology Program, Cancer Biology, College of Veterinary Medicine and Biomedical Sciences, Colorado State University, Fort Collins, Colorado, United States of America; 3 Department of Radiation Oncology, Duke University Medical Center, Durham, North Carolina, United States of America; 4 Cancer Center Metabolomics Core, Anschutz Medical Center, University of Colorado Denver, Aurora, Colorado, United States of America; 5 Department of Surgery, Duke University Medical Center, Durham, North Carolina, United States of America; 6 Department of Pathology, Duke University Medical Center, Durham, North Carolina, United States of America; 7 The Preston Robert Tisch Brain Tumor Center, Duke University Medical Center, Durham, North Carolina, United States of America; 8 Department of Cell Biology, Duke University Medical Center, Durham, North Carolina, United States of America; 9 Department of Anesthesiology, Anschutz Medical Center, University of Colorado Denver, Aurora, Colorado, United States of America; Beijing Tiantan Hospital, Capital Medical University, China

## Abstract

The unfolded protein response (UPR) is an endoplasmic reticulum (ER)-based cytoprotective mechanism acting to prevent pathologies accompanying protein aggregation. It is frequently active in tumors, but relatively unstudied in gliomas. We hypothesized that UPR stress effects on glioma cells might protect tumors from additional exogenous stress (ie, chemotherapeutics), postulating that protection was concurrent with altered tumor cell metabolism. Using human brain tumor cell lines, xenograft tumors, human samples and gene expression databases, we determined molecular features of glioma cell UPR induction/activation, and here report a detailed analysis of UPR transcriptional/translational/metabolic responses. Immunohistochemistry, Western and Northern blots identified elevated levels of UPR transcription factors and downstream ER chaperone targets in gliomas. Microarray profiling revealed distinct regulation of stress responses between xenograft tumors and parent cell lines, with gene ontology and network analyses linking gene expression to cell survival and metabolic processes. Human glioma samples were examined for levels of the ER chaperone GRP94 by immunohistochemistry and for other UPR components by Western blotting. Gene and protein expression data from patient gliomas correlated poor patient prognoses with increased expression of ER chaperones, UPR target genes, and metabolic enzymes (glycolysis and lipogenesis). NMR-based metabolomic studies revealed increased metabolic outputs in glucose uptake with elevated glycolytic activity as well as increased phospholipid turnover. Elevated levels of amino acids, antioxidants, and cholesterol were also evident upon UPR stress; in particular, recurrent tumors had overall higher lipid outputs and elevated specific UPR arms. Clonogenicity studies following temozolomide treatment of stressed or unstressed cells demonstrated UPR-induced chemoresistance. Our data characterize the UPR in glioma cells and human tumors, and link the UPR to chemoresistance possibly via enhanced metabolism. Given the role of the UPR in the balance between cell survival and apoptosis, targeting the UPR and/or controlling metabolic activity may prove beneficial for malignant glioma therapeutics.

## Introduction

Malignant gliomas are highly lethal and devastating diseases that eventually fail to respond to current therapies. The present standard of care (maximal surgical resection, external beam radiation concurrent with adjuvant temozolomide chemotherapy) for the most aggressive forms of the disease results in a median survival of less than 15 months post-diagnosis [1], and this figure has changed little in the past 20 years [2]. These tumors are highly invasive [3,4], indicating an active extracellular microenvironment; they are also highly chemo- and radio-resistant [5–7] indicating elevated stress responses against internal (ie, metabolic) and external insults [8–10]. The devastating consequences of glioma biology may be enabled by the unfolded protein response (UPR), which can both support secretory pathway function and promote stress resistance via altered metabolism [11–13].

Enhanced expression of endoplasmic reticulum (ER) chaperones occurs in response to activation of the UPR, a cytoprotective pathway designed to relieve cellular stress resulting from increased biosynthetic demands [14]. Although initially identified as a biologically elegant quality control mechanism against aberrantly folded proteins in the ER lumen, recent discoveries have demonstrated that the UPR can regulate cell fate and apoptosis. The UPR is divided into two coordinately regulated responses: (1) an initial attenuation of global protein synthesis to slow the influx of newly synthesized proteins into the secretory pathway, and (2) a transcriptional remodeling event that elevates expression of a cohort of stress response genes [14]. In biological contexts, these downstream events may be provoked simultaneously or may be individually activated. In mammalian cells, the UPR is comprised of activators and effectors. Three ER transmembrane molecules—IRE1, PERK and ATF6—function as individual activators of transcriptional (IRE1, ATF6) and translational (PERK) programs. The combined activities of PERK, IRE1, and ATF6 yield increased production of effector stress-response (XBP-1, ATF4, ATF6) and pro-apoptotic (CHOP/GADD153) transcription factors, in addition to enhanced expression of ER-resident chaperones, such as BiP/GRP78 and GRP94 [14]. Thus, the UPR pushes cells to either “work through” the problem—leading to recovery from the stress--or the cells undergo apoptosis if the stress is insurmountable.

The UPR or elements of it (eg, BiP/GRP78) have been associated with reduced responses to cancer chemotherapy [15–17]. Chemoresistance is also correlated with hypoxic signaling and elevated aerobic glycolysis (the “Warburg effect”) in order to maintain sufficient intracellular ATP levels [18]. Hypoxia also correlates with UPR activation [19–21] where protein markers of each overlap (eg, Tribbles homolog 3 [22]). Thus, there is an intersection of tumor stress, chemoresistance, and metabolic upregulation in the UPR.

UPR transducers and activators are themselves responsive to nutritional states, modulating lipogenesis [23]; in particular, induction of the UPR leads to phospholipid biosynthesis necessary for the expansion of the ER membrane, for passage of proteins thru the secretory pathway [24]. This holds especially true for cell types with high secretory capacities such as B cells [25]. However, there are relatively few connections made between the unfolded protein response and glycolysis [26], even though relationships between glycolysis, lipogenesis [27], and other intermediate metabolites have been known for some time [28]. The recent advances in nuclear magnetic resonance (NMR) based metabolomics allowed for metabolic characterization of various brain tumor classes [29]; however, this technique has yet to be applied for the metabolic assessment of the UPR stimulation in brain tumors.

Elevated chaperone protein levels are evident in brain tumor cells, xenograft tumors, and patient samples [30–32], including elevation and surface localization of BiP/GRP78. Others have identified GRP78 as a potential chemosensitizer in malignant gliomas [33], as well as a role for IRE1 in angiogenesis and tumor cell invasion in a glioma model. From these studies we postulated that expression of other ER chaperones and upstream UPR transcription factors may also be enhanced in gliomas as evidence of an inducible or “chronically” activated UPR. The UPR is active in many tumor types [34,35] including gliomas [36–39] but in general little is known about the global characterization of the UPR in gliomas. Our objective here was to perform a detailed characterization of UPR-mediated transcriptional targets and translational regulation in human malignant gliomas, cell lines, and xenograft tumors to evaluate the UPR activation status. We identified the classic transcriptional components of the UPR in human glioma cell lines and in paired solid xenograft glioma tumors, along with the maintenance of cellular processes despite the continuing UPR stress. To examine the translational components of UPR function, we scrutinized gene expression data of polyribosome-bound mRNA of xenograft tumors and parent cell lines, whereupon we demonstrated that the UPR drives profound chemoresitance when induced in glioma cell lines. Analyses of existing human patient glioma cDNA microarray data revealed that elevated levels of UPR transcription factors and ER chaperones correlated with poor patient prognosis; Western blots of high grade gliomas and tissue microarray immunohistochemistry verified high expression of UPR players, especially GRP94, in high grade gliomas. Gene ontology and pathway analyses led to NMR metabolomic studies showing a generalized activation of cell metabolism in UPR-induced glioma cells with profiles reflected in patient tumors, including differential outputs between primary and recurrent tumors. We conclude that activation of the UPR signaling pathways is a prominent feature of glioma biology that leads to metabolic shifts and enhances chemoresistant features of gliomas, which has implications for therapeutic interventions.

## Results

### Malignant glioma xenograft tumors and cell lines exhibit UPR activation: mRNA and protein levels

**Figure 1:**
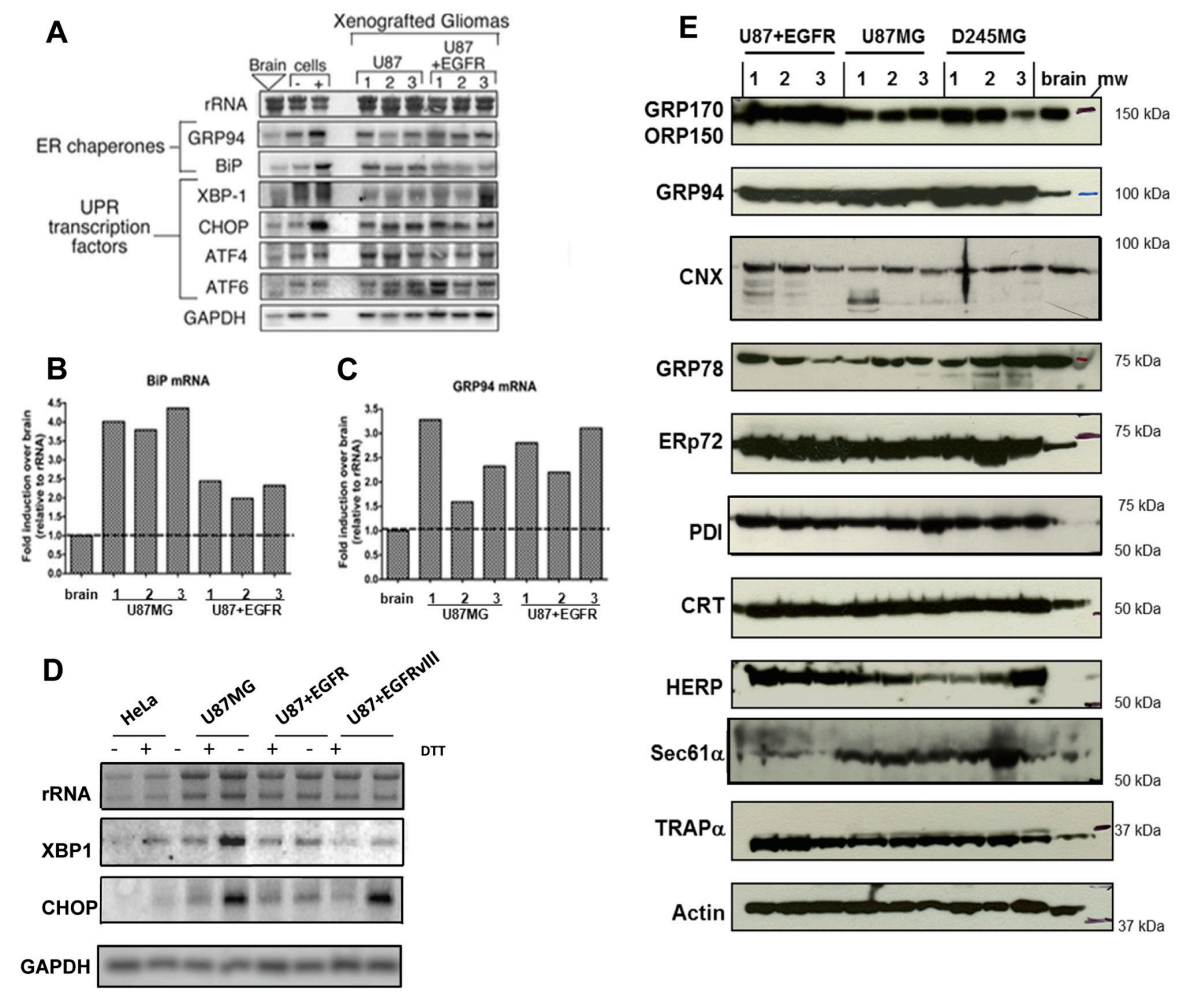
Identification of UPR signaling response patterns in high-grade glioma xenografts and cell lines. Human glioma xenografts grown in *nu/nu* mice were derived from U87MG, and U87+EGFR (wild type) (cell lines described in the text and Materials and Methods). (**A**) Northern blots of 10 µg total RNA from replicate tumors (n=3) and normal brain from *nu/nu* mice; 10 µg total RNA from U87 tissue culture cells (“cells”) treated with the reducing agent DTT ([+]) lanes) to induce the UPR. Note transcriptional upregulation of UPR-induced mRNAs for ER chaperones (GRP94, BiP/GRP78) and UPR signaling components (XBP-1, CHOP, ATF4, ATF6). Quantification of BiP/GRP78 (**B**) and GRP94 (**C**) mRNA expression compared to mean level of expression in normal murine brain (dotted line). (**D**) U87MG, U87+EGFR, and U87+EGFRvIII (U87 cells transfected with the tumor-specific EGFR mutant variant III [in-frame deletion of exons 2-7]) cells show greater UPR inducibility with 1 mM DTT (determined by Northern blotting for XBP-1 and CHOP messages) than do HeLa cells. (**E**) Human glioma xenografts were derived from U87MG, U87+EGFR, and from D245MG, from a patient-derived Duke high grade glioma (from the Duke Brain Tumor BioRepository). Immunoblot of replicate tumors (n=3) from xenograft glioma models and normal brain from *nu/nu* mice. Note upregulation of ER chaperones in tumor lysates vs brain lysates: GRP170/ORP150, GRP94, calnexin (CNX), ERp72, protein disulfide isomerase (PDI), calreticulin (CRT), homocysteine-induced ER protein (HERP), and ER membrane markers (Sec61α and translocon associated protein, TRAPα) relative to loading control (β-actin). GRP78/BiP protein expression was variable in our Western blot assays. Blots probing for actin as loading controls are found in Supplemental Figure S1. Blots for GRPs 170 and 78, for ERp72 and TRAPα were replicate blots. Blots for GRP94, CNX, CRT, HERP, and Sec61α were stripped and reprobed for actin.

To assess UPR activation in glial tumors, glioma cell lines and their corresponding xenograft tumor samples were examined for relative mRNA levels for ER chaperones and UPR-coupled transcription factors. Cell lines/tumors examined included U87MG, an established (human) patient-derived glioma line and U87+EGFR, which are parent U87MG cells stably overexpressing the wild type EGFR receptor (also referred to as U87-wtt); EGFR overexpression is considered a hallmark of gliomas [40], and this cell line is a popular model for that situation. In addition, xenograft tumors were compared to proliferating glioma cells in culture to determine if the UPR activation profiles of cultured glioma cells and cohort solid tumors differed. The transcriptional profiles of the human glioma lines were examined both under steady state conditions and following UPR induction with the ER stress agent dithiothreitol (1 mM DTT, 4 hrs; “+” lanes). In these experiments, total RNA from three representative tumors of each xenograft type were analyzed by Northern blot (Figure 1A). Consistent with our expectations, transcripts for the ER chaperones GRP94 and GRP78 were upregulated in the U87MG and U87+EGFR gliomas, relative to normal mouse brain from healthy animals (Figure 1A). Quantification of mRNA expression, normalized to rRNA levels, revealed a maximal 3- and 4.5-fold increase in message levels for GRP94 and GRP78, respectively (Figure 1B, C).

Levels of key UPR transcripts XBP-1, CHOP, ATF4, and ATF6 were only nominally detected in normal brain and unstressed U87 glioma cells (Figure 1A), as reported by other studies [41]. In contrast, we observed elevated expression of UPR transcription factors in all xenograft tumor models examined, with results from U87MG and U87MG+EGFR xenografts shown in Figure 1A. Relative to normal mouse brain, xenograft tumors displayed increased levels of XBP-1 (up to 2-fold increase), CHOP (up to 3-fold increase), ATF4 (up to 2-fold increase), and ATF6 (up to 2.7-fold increase) mRNAs. In glioma cultures undergoing acute pharmacological UPR induction by DTT treatment, these transcription factors show further increased expression. Here, the levels of UPR transcription factor messages present in the solid xenograft tumors mirror the profiles seen in pharmacologically induced cells, illustrating robust (and perhaps chronic) activation of the UPR signal transduction pathway in malignant glioma xenografts.

The inducibility of the UPR in DTT-treated U87MG cells (and those expressing EGFR or the oncogenic mutated EGFRvIII, which is an in-frame deletion mutant removing 801 base pairs of exons 2-7 [42], yielding a constitutively activated receptor) led us to compare those cells to another tumor cell line, HeLa, in terms of response the UPR inducer DTT. As shown in Figure 1 D, U87MG and variants (including lines expressing the mutated EGFRvIII) increased mRNA expression of XBP-1 1.2 to 1.5 times over that of HeLa, and CHOP expression increased to almost 3-fold compared to DTT-treated HeLa cells. Thus, the U87 cell lines show highly inducible UPR-related transcripts compared to another tumor line as well as upregulated transcripts compared to normal mouse brain

The upregulation of ER chaperone proteins and UPR effectors is also evident in human glioma xenografts. The transcriptional activation of various UPR response genes (Figure 1A–D) implied that other ER chaperones may be present at elevated levels in glioma, leading us to examine expression patterns of a panel of ER molecular chaperones in human glioma xenograft tumors compared with normal mouse brain. The data depicted in Figure 1E were obtained from serially passaged solid tumors originally generated in *nu/nu* mice via subcutaneous injection of cells derived from human high-grade gliomas. These models include the aforementioned U87MG and U87+EGFR, and D245MG, from the Duke University, Brain Tumor BioRepository collection (D245MG does not grow in cell culture, and serves as an example of tumors that only grow as xenografts). Using independent tumor samples derived from individual mice, immunoblot analysis identified a 3-5 fold up-regulation of the ER lumenal chaperone GRP94, relative to normal murine brain tissue, in all three glioma models (Figure 1E). Consistent with the data obtained for GRP94, levels of other ER resident chaperones were also substantially elevated, including ERp72, a PDI-family member; PDI (protein disulfide isomerase) itself; the lectin-binding chaperone, calreticulin; and HERP (Homocysteine-responsive endoplasmic reticulum-resident), a transmembrane, ubiquitin-like protein. Chaperone-related proteins showing variable expression (in some cases, lower levels) compared to normal mouse brain include ORP150/GRP170, the hypoxia-induced, 150-kDa oxygen-regulated protein; calnexin, which works coordinately with calreticulin as a chaperone (and has multiple potential post-translational modifications [43]), and GRP78/BiP, which in our hands did not show dramatic increased protein expression in xenograft samples compared to brain, at least in immunoblots (however, see Figure S1 and later figures). Furthermore, we observed an increase in the expression of ER translocon members, including the translocon-associated protein α subunit (TRAPα) and Sec61α, the latter being upregulated in most of the tumor samples. Thus, both lumenal and membrane proteins of the ER are upregulated in these glioma xenografts, indicative of the UPR and of an active secretory profile in these tumors. Actin loading control blots are shown in Supplemental Figure S1.

We confirmed upregulation of these ER chaperone proteins by immunohistochemical staining on formalin-fixed, paraffin-embedded xenograft sections. Representative micrographs are shown in Figure S2 and demonstrate that relative to a typical normal murine brain section (cortex), the tumor tissues exhibit elevated levels of the ER chaperones GRP94, GRP78, ERp72, calreticulin, PDI, and HERP, while the stress-induced ORP150/GRP170 and calnexin show variable staining relative to brain. These data recapitulate the immunoblot results in Figure 1E and are consistent with a broad and sustained up-regulation of ER chaperone expression in the tumor models.

**Figure 2:**
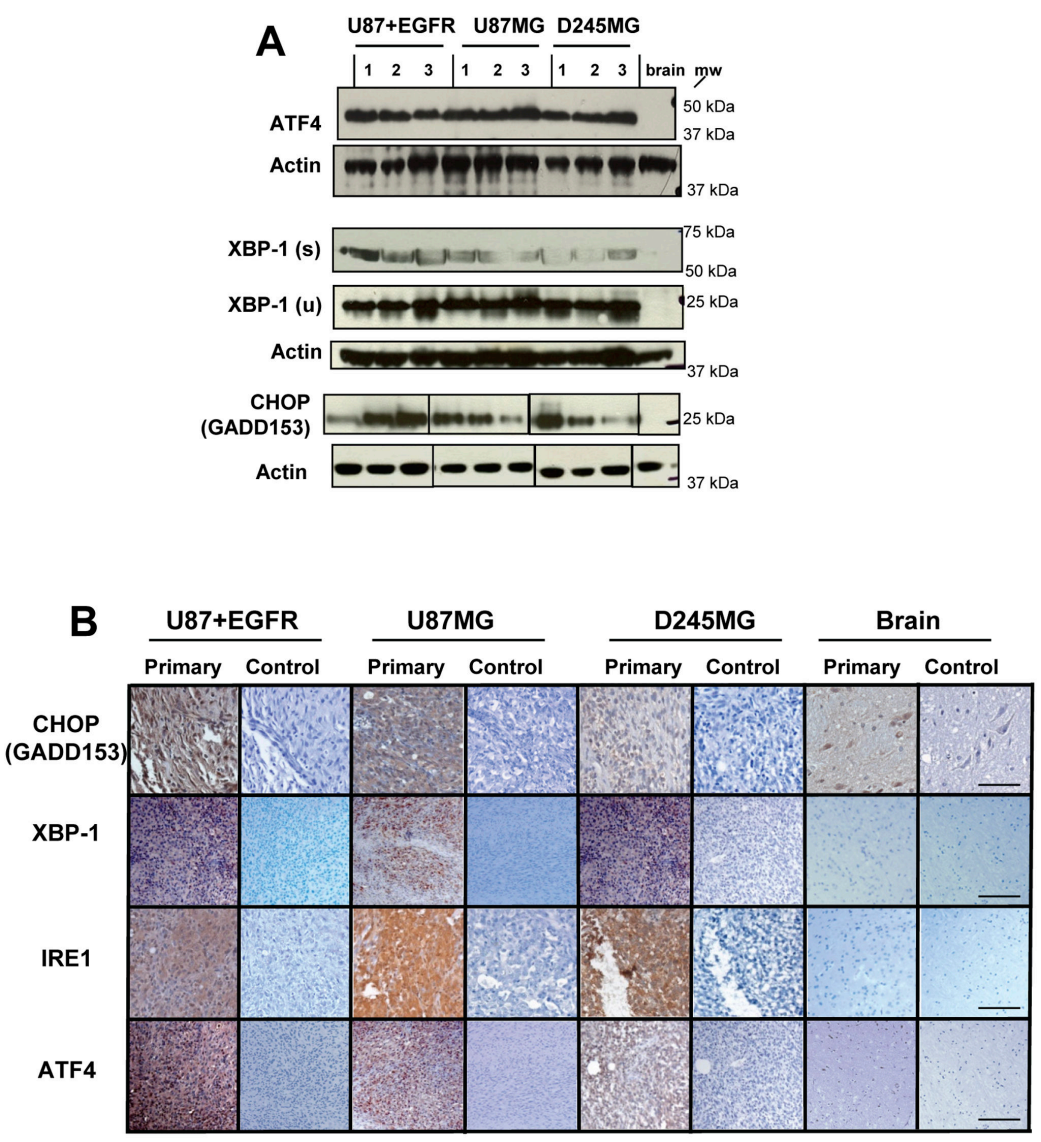
UPR effectors from all arms of UPR signaling are upregulated in glioma xenograft tumors. (**A**) The same xenograft samples used in Figure 1 were immunoblotted with antibodies against ATF4, XBP-1 (spliced [s]—ie, active form, and unspliced [u]), CHOP/GADD153, and actin as a loading control (re-probed after stripping blots; actin for ATF4 was a replicate blot). While CHOP expression was variable, the rest of the UPR effectors were substantially upregulated in their expression compared to normal brain. (**B**) Representative immunohistochemical staining from paraffin-embedded, formaldehyde-fixed tissue sections of normal brain from *nu/nu* mice and xenografts from U87+EGFR, U8MG7, and D245MG samples. Control panels are probed with a species-matched irrelevant antibody at concentrations identical to the experimental/primary antibody. The scale bar represents 100 µm. Slices are closely matched in sequence for a given tumor sample; the tumors used here are not the same as those used for the Western blots in (**A**).

The protein expression of UPR effectors (ATF4, XBP-1, CHOP/GADD153) is also increased in glioma tumors, indicating that their enhanced transcription indeed leads to increased protein levels. The immunoblots in Figure 2A show that ATF4, the spliced (active) and unspliced versions of XBP-1, and CHOP demonstrate dramatically increased expression compared to normal murine brain. This is again reflected in immunohistochemistry of glioma xenograft tumors (Figure 2B); high levels of CHOP and ATF4 (driven by the PERK arm of the UPR) are evident compared to normal brain, as are those of XBP-1 (where we cannot distinguish between spliced and unspliced versions with this antibody). High levels of IRE1 suggest its activation, and this is validated by evidence of the spliced forms of XBP-1.

### The UPR is inducible in fresh primary cultures from newly-resected glioblastomas

**Figure 3:**
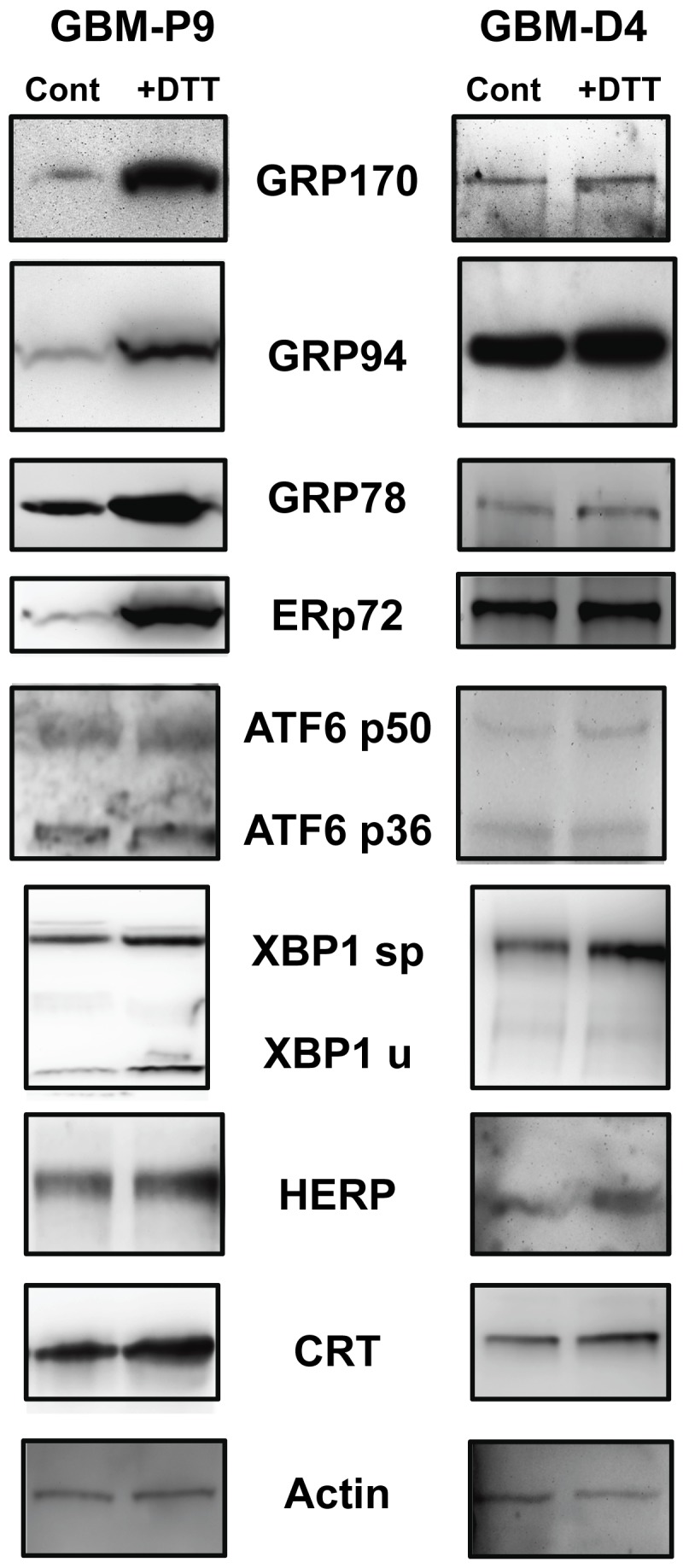
Primary tissue culture cells from newly-resected gliomas also display inducible elements of the UPR. Dissociated cell cultured from freshly-resected GBMs were grown under serum-free conditions and were treated (or not, “Cont”) with 1 mM DTT (“+DTT”) for 4 hrs. Cell cultures were harvested, and cells lysed described. Proteins were separated on SDS-PAGE and Western blotted and probed with the antibodies listed. Upregulation of some of the UPR components is evident. Actin probe is used as a loading control, from the stripped CRT blot. Other actin blots to verify loading are shown in Supplemental Figure S3.

From Figures 1 and 2 it is evident that the UPR is inducible in established tumor lines, including those of increasing aggressiveness (ie, the U87 lines transfected with EGFR and EGFRvIII). UPR components are also present in transplantable xenograft tumors at levels frequently higher than in unaffected brain; nonetheless, a question remains if such inducibility is possible in very low passage GBM primary cultures from newly surgically-resected tumors. In such situations the heterogeneity of the sample may impact UPR inducibility *ex vivo*. To test this, we prepared dissociated cell cultures from freshly harvested patient GBM in serum-free medium and treated the cultures with DTT or left the cultures untreated as controls. As seen in Figure 3, many UPR-related protein components are upregulated following DTT treatment (eg, GRPs 170 and 94, XBP-1, and HERP) but not all members are (eg, ATF6 forms), and not necessarily for both tumors (GRP78, ERp72). These results do suggest that even in cultures from freshly-resected, highly heterogeneous tumor cell populations, there is some level of UPR inducibility (we should point out that these experiments were conducted within two weeks of putting the dissociated cells in culture). Blots probed with actin antibodies as loading controls are shown in Supplemental Figure S3.

### Induction of the UPR transcriptional program by cell stress agents in glioma cell culture

**Figure 4:**
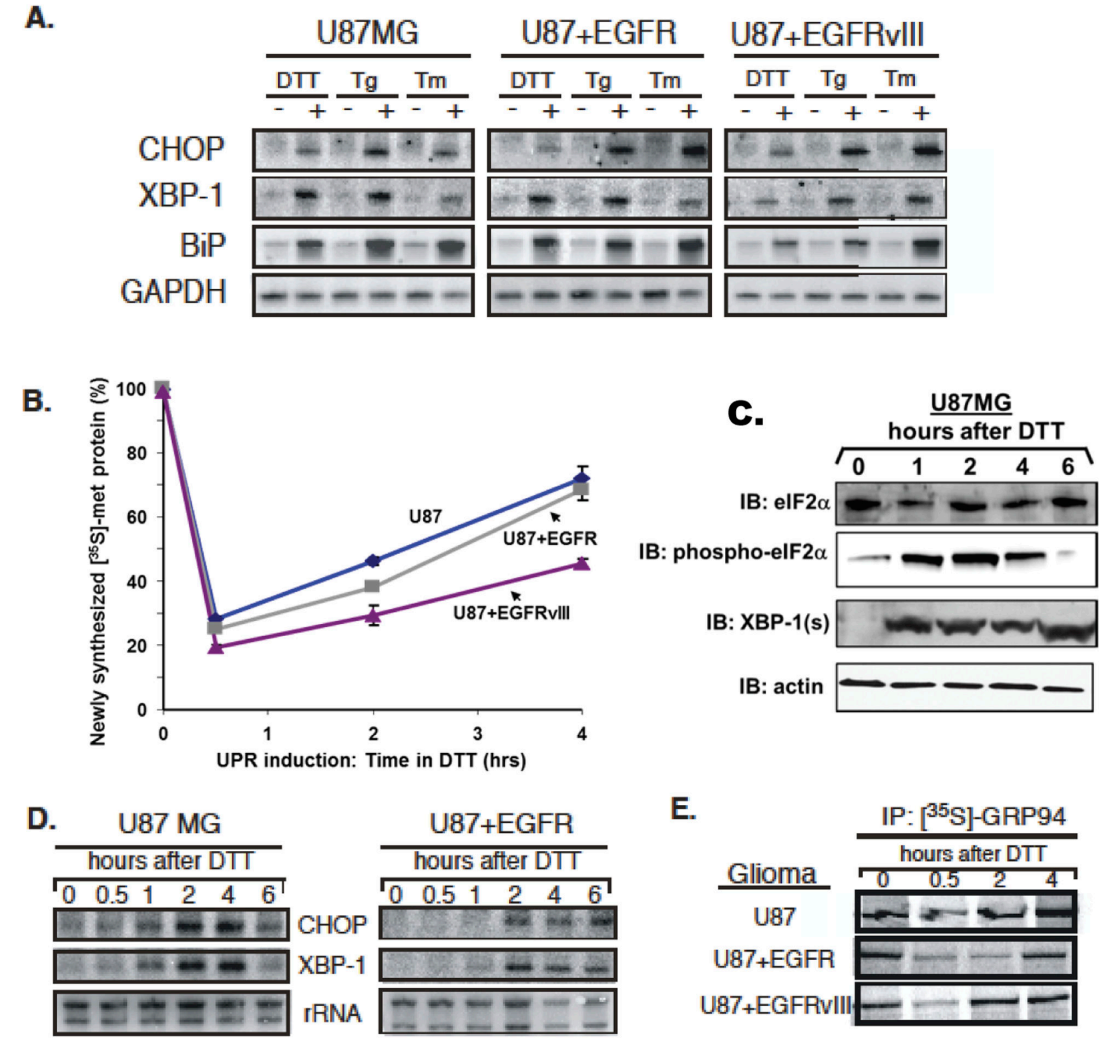
Human glioma cells respond to persistent ER stress with UPR induction and rapid recovery of protein synthesis. Human glioma tissue culture models examined include: U87MG, U87+EGFR, and U87+EGFRvIII, from U87 parent lines stably transfected with a constitutively-active oncogenic EGFR and the extracellulary truncated EGFR variant III. (**A**) Northern blot analysis of human glioma tissue culture cells after a 4-hour treatment with the following pharmacological inducers of the UPR: 1mM dithiothreitol (DTT), 0.5 µM thapsigarin (Tg), or 2.5µg/ml tunicamycin(TM). Blots were probed for message levels of CHOP, XBP-1, GRP78/BiP, and GAPDH. (**B**) Levels of total newly-synthesized protein from 0–4 hours after a 1mM DTT treatment were assayed as TCA precipitable [^35^S]-methionine-labeled protein. Protein synthesis rapidly declined and then rapidly recovered despite the presence of reducing agent in the culture. (**C**) The time course of eIF2α phosphorylation (“phospho- eIF2α”) was followed by immunoblotting during DTT treatment of U87MG cells, as was induction of spliced XBP-1 (“XBP-1(s)”. The actin loading control blot is a replicate blot. Following a 0-6 hour 1mM DTT treatment in U87 cell culture, (**D**) RNA was analyzed by Northern blot in a kinetic analysis of CHOP and XBP-1 mRNA. (**E**) Glioma cell cultures were labeled with [^35^S]-methionine during a 0-4 hour 1mM DTT treatment. GRP94 was immunoprecipitated to detect newly-synthesized protein.

UPR induction can occur in response to the loss of ER oxidative capacity, decreases in ER lumenal calcium levels and/or disruptions in N-linked glycosylation [14]; alternate forms of ER stress can also provoke distinct UPR responses [44]. At present, however, little is known regarding the ER stress sensing and coincident UPR signaling capabilities of human gliomas. Because this question cannot be readily determined in xenograft tumors *in situ*, we have addressed this question in cell culture, using well-defined pharmacological treatments and three human glioma lines: U87MG, U87+EGFR, and U87+EGFRvIII [42,45,46]. Following an acute 4 hour treatment with the reducing agent DTT, all glioma lines demonstrated a marked induction of XBP-1, BiP/GRP78, and CHOP mRNAs, while maintaining steady levels of GAPDH message (Figure 4A). Similar trends of UPR induction were observed with distinct pharmacological stresses: thapsigargin, an inhibitor of the ER Ca^2+^-ATPase, and tunicamycin, an inhibitor of N-linked glycosylation (Figure 4A). These data demonstrate that human glioma lines, differing in EGFR expression, display robust UPR activation in response to these known acute UPR-inducing disruptions in ER physiology. We also examined UPR-related protein outputs by Western blotting in the U87MG cell line and the GBM-P9 primary culture model following tunicamycin or thapsigargin treatment (Supplemental Figure S4). Both UPR inducers led to increased expression of most of the chaperones studied (eg, GRPs 94 and 78, ERp72, CRT, and HERP), with variable changes in CHOP/GADD153, and minimal changes in XBP-1, where there was already substantial XBP-1 spliced protein form present. Thus, these commonly-used inducers of the UPR [47] led to increased expression of UPR messages and proteins in multiple cell types. Since dithiothreitol treatment is known to be quite specific for UPR induction [48] and drove consistent upregulation of UPR components, we utilized it for the remainder of the studies.

To examine UPR-elicited translational suppression following acute ER stress, total protein production was examined in glioma cell cultures during a comparable 4-hour time course of DTT treatment (Figure 4B). As depicted, by 30 minutes of DTT treatment total protein synthesis activity decreased by ~80% in all cell lines examined, followed by a recovery over the ensuing 4 hours. Immunoblot analysis demonstrated a coordinate increase in phospho-eIF2α levels, which were maximal at 30-60 minutes, followed by subsequent decline in concert with the recovery of protein synthesis activity (Figure 4C). Also evident in the cell line models was the rapid induction of the active form of XBP-1 (Figure 4C). The remarkable recovery of global protein synthesis amidst redox stress seen in the acute tissue culture UPR induction models may be illustrative of the *in vivo* biology of glioma, where high rates of cell growth and division are maintained although there is compelling experimental evidence of chronic hypoxia/anoxia [49,50].

In companion studies, the time course of ER stress-dependent transcriptional induction of the UPR transcriptional program was assayed. Following DTT treatment, an accumulation of both CHOP and XBP-1 mRNAs was observed in U87MG and U87+EGFR cultures (Figure 4D). Interestingly, mRNA levels for both transcription factors began to diminish towards basal levels by 6 hours, signifying a reversible response, in contrast to tumors, which displayed constitutive levels of markers (Figure 1A). Also, the level of spliced XBP-1 is maintained for at least 6 hrs after induction (Figure 4C) despite the diminution of message. As a downstream product of XBP-1(s) signaling, we examined levels of newly synthesized ER chaperone protein, GRP94 and demonstrated a coordinate decrease at 30 minutes post-treatment with recovery by 4 hours (Figure 4E).

### Polyribosomal RNA expression profiling reveals tumor-specific stress responses

**Figure 5:**
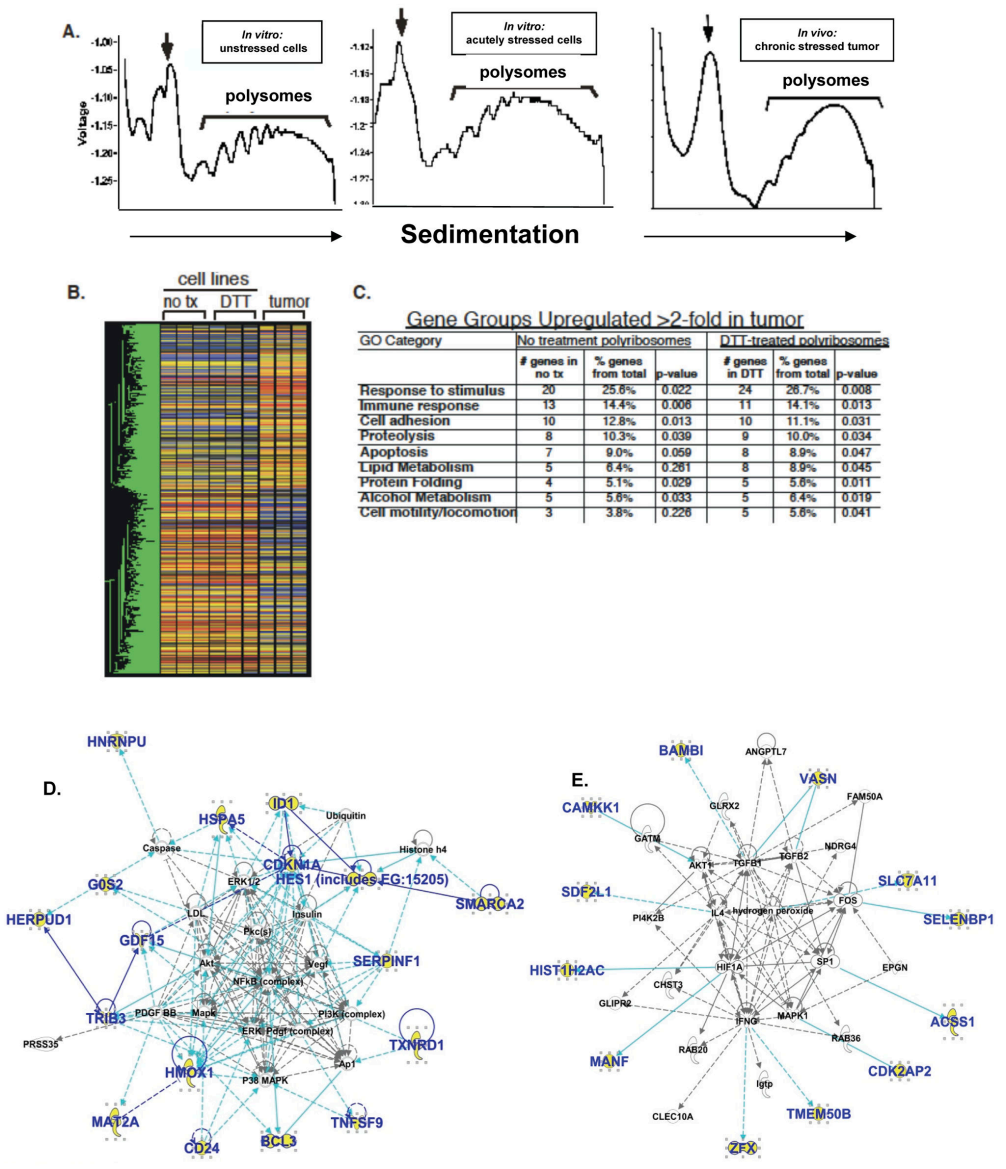
cDNA microarray analysis of polyribosome-engaged transcripts in control and UPR-activated cells and xenograft glioma tumors. (**A**) Polyribosome traces from U87MG glioma models experiencing (or not) various stresses: unstressed cells, DTT-treated (acutely) stressed cells, and xenograft tumor-derived samples (“*in vivo* chronic stressed tumor”). Each sample was analyzed in triplicate. Following homogenization, sample lysates were layered over a linear sucrose gradient (15-50%), separated at 150,000X g for 3 hours and processed as described (Figure S2). Polyribosome-containing regions were pooled, total RNA extracted and submitted to the Duke Microarray Facility for analysis. (**B**) Heatmap of triplicate samples with one-way ANOVA analysis (P<0.01). Note the major distinctions between solid tumor polysome mRNAs and those of the cells in culture; over 1000 genes were differentially recruited to polysomes in solid tumors compared to tissue culture cells. (**C**) From the one-way ANOVA, p<0.01, genes with 2 of 3 replicates with a p<0.05 were selected. Listed here are GO groups and accompanying numbers of genes (GeneSpring) upregulated in tumor samples (> 2-fold, p<0.01) over either stressed or unstressed cells, with 5% or more of the total genes represented in the tumor. (**D** and **E**) Interactomes deduced from the 30 overexpressed genes sorted via Ingenuity Pathway Analysis; genes from the entry set are in bold blue; solid lines indicate direct connections between gene products, while dashed lines are indirect connections. Top Network/Associated Network Function in **D** is “Cell Death, Gene Expression, Free Radical Scavenging” with a network score of 44. Top Network/Associated Network Function in **E** is “Gene Expression, Cellular Development, Cell Death”, score = 27. The scores are –log (p values) (Fisher Exact Test).

The data presented above demonstrate a robust UPR program in human glioblastoma cell lines along with a chronic response in solid glial tumors. In cell lines, levels of UPR-induced transcription factors resolve to near pre-induction levels in about 6 hours, demonstrating the transient nature of UPR activation in cell culture. However, data from xenograft tumors suggests sustained activation of the UPR, raising the likelihood that the transcriptional output of cultured human glioma cell lines differs from that present in xenograft tumors (however, the downstream effects, such as reduced overall protein synthesis and subsequent apoptosis, do appear uncoupled from UPR activation). Indeed, studies have demonstrated that transcriptional patterns of *in vivo* solid tumors can differ substantially from their parent cell lines [51,52]. We have undertaken polysome analyses for mRNA expression profiling under various cellular/tissue conditions. Polysomes (polyribosmes) are clusters of ribosomes bound to mRNA molecules, essentially at the point of protein translation. Unlike our transcriptional studies (eg. Figures 1 and 4), and most gene expression analyses, where any mRNA may be included (no matter what its fate), polysome mRNAs are destined for translation, and those messages with higher quantities of ribosomes (ie, heavier polysomes) are presumed to be purposefully highly translated to increase protein expression. We have shown UPR-related mRNA loading onto glioma xenograft-derived tumor polyribosomes where GRP94, BiP/GRP78 and GAPDH mRNA distributions in the gradient fractions clearly demonstrate the efficient recruitment of UPR-sensitive transcripts into the heavy-sedimenting polyribosome fractions (Supplemental Figure S5). The arrows point to the 80S monosome (ie, mRNAs bound by a single ribosome), with subsequent peaks of 2, 3, 4, 5 etc ribosomes going to the right. Messages that are likely differentially translated at a higher rate will be in the fractions towards the right of the trace. To further investigate the role of translational control between *in vivo* (tumor) and *in vitro* (cell culture) cancer models, we performed cDNA microarray analysis of polyribosome-associated RNA from differing UPR-glioma models. We compared (1) unstressed U87 cells in culture, (2) acutely stressed (DTT-treated) U87 cells in culture, and (3) U87 xenograft tumors experiencing chronic tumor-associated stress. By refining our analysis to transcripts recruited onto polyribosomes, we examined global remodeling of the cellular translation state, a primary mechanism of cellular stress survival, at a point close to protein translation. Here we again examined polyribosomes by velocity sedimentation through linear sucrose gradients (Figure 5A). mRNAs co-sedimenting with heavy polyribosomes were isolated and hybridized to a human 37,000 gene oligonucleotide array (Duke University Microarray Facility). There is a noticeable shift to heavier sedimentation overall in the DTT-stressed cells compared to those in the unstressed U87 cells (Figure 5A), again, despite the general tendency in cells to reduce translational output during UPR stress.

Following one-way ANOVA analysis (p<0.01, 1085 genes), a distinct network of polysome-bound transcripts was revealed in the solid tumor relative to the parent cell culture. Heat map analysis (Figure 5B) illustrates the magnitude of this inductive phenomenon in the solid tumor model compared to either DTT-stressed (UPR) or unstressed cell culture models. In contrast to the gross changes in the tumor translation state, acute induction of UPR via DTT treatment in cell lines induced changes in only a small subset of genes, as illustrated (Figure 5B). Of the 1085 recruited transcripts from solid tumors identified by one-way ANOVA, a subset of 30 genes was upregulated 2-fold or more in the DTT-treated cells compared with the untreated cells (Table 1, functional analysis in Table 2), illustrating the specificity of DTT treatment for activation of the canonical cell culture model of the UPR. To further explore these differences between solid tumors and treated/untreated cells, in Figure 5C we identified gene groups based on polysome-associated transcripts from the *in vivo*-grown tumors that were present at greater than 2-fold compared to polysome mRNAs from acutely-stressed cell culture and the non-stressed cell culture mRNAs. Using gene ontogeny (GO) analysis of those mRNAs enriched at least 2-fold in the tumor samples, we observed up-regulation of GO groups involved in the immune response, response to stimulus, cell adhesion, and cell motility. Additionally, we identified elevated levels of components associated with specific biosynthetic events, including proteolysis pathways, lipid metabolism, protein folding and alcohol metabolism (the latter associated with the URP [53]). These processes are critical to tumor establishment, defense, proliferation, and invasion/migration, hallmarks of high grade gliomas.

**Table 1:** Genes in polyribosome fractions upregulated > 2-fold in DTT-treated U87MG cells over untreated cells.

**UniGene ID**	**Fold Change**	**Gene Name**
Hs. 250666	6.108	Transcription factor HES-1 (Hairy and enhancer of split 1)
Hs. 632460	4.045	Selenium binding protein 1
Hs. 694727	3.882	Serpin-F1 /PEDF/EPC-1 (pigment epithelium-derived factor)
Hs. 390594	3.638	Cystine/glutamate transporter, solute carrier family 7 member 11
Hs. 433668	3.473	Transmembrane protein 50B (HCV p7-trans-regulated protein 3)
Hs. 436446	3.417	ARMET protein (arginine-rich, mutated in early stage tumors)
Hs. 533336	3.221	BMP and activin membrane-bound inhibitor homolog (BAMBI)
Hs. 303116	3.136	Stromal cell-derived factor 2-like (SDF2 like protein 1)
Hs. 605502	3.132	BiP (glucose regulated protein 78 kDa)
Hs. 336681	3.007	Zinc finger protein, X-linked
Hs. 616962	2.877	Growth differentiation factor 15 / Macrophage Inhibitory Cytokine-1
Hs. 504609	2.587	Inhibitor of DNA binding 1, dominant negative HLH protein
Hs. 166463	2.564	Heterogeneous nuclear ribonucleoprotein U
Hs. 372579	2.436	Mental retardation X-linked 85/Vasorin/SLIT2
Hs. 1524	2.388	Tumor necrosis factor (ligand) superfamily member 9
Hs. 408542	2.341	Sugen kinase 493/ hypothetical protein BC007901
Hs. 567352	2.301	Thioredoxin reductase 1
Hs. 146393	2.210	HERP (homocysteine-responsive ER-resident ubiquitin-like protein
Hs. 516157	2.193	Methionine adenosyltransferase II alpha
Hs. 529353	2.146	Acyl-CoA synthetase short-chain family member 1
Hs. 652291	2.130	CD24 molecule
Hs. 517581	2.122	Heme oxygenase 1 (EC 1.1499.3)
Hs. 31210	2.095	B-cell lymphoma 3-encoded protein (Bcl-3 protein)
Hs. 484950	2.069	Histone 1 H2ac
Hs. 516862	2.053	Tribbles homolog 3
Hs. 8417	2.041	Calcium/calmodulin-dependent protein kinase kinase 1
Hs. 298990	2.037	Probable global transcription activator SNF2L2/SMARCA2
Hs. 523835	2.033	Cyclin-dependent kinase 2 associated protein 2 (DOC 1R)
Hs. 370771	2.028	Cyclin-dependent kinase inhibitor 1 (p21) (CDK-interacting protein 1)
Hs. 432132	2.020	G0/G1switch 2

Of the 1085 genes significantly over-expressed in solid U87 tumors compared to U87 tissue culture cells, these 30 genes were expressed > 2-fold in DTT-treated U87 cells compared to untreated U87 cells. UniGene Human (Homo sapien, “Hs”) identifiers are listed at left.

**Table 2:** Functional grouping of the 30 genes upregulated ≥ 2-fold in U87MG cells treated with the UPR inducer DTT.

**Metabolic/ER Stress and UPR-related**	
**Gene/Protein Name**	**Selected References**
cystine/glutamate transporter	[124]
ARMET	[125]
SDF-like protein 1	[126]
BiP/GRP78	
thioredoxin reductase 1	[127]
HERP	
methionine adensosyltransferase II alpha	[128]
heme oxygenase 1	[129]
Tribbles homolog 3	[130]
calcium/calmodulin-dependent protein kinase kinase 1	[131]
G0S2	[132]
**Transcriptional Regulation**	
**Gene/Protein Name**	**Selected References**
HES-1	[133]
ID-1	[134]
hnRNPU	[135]
Bcl-3	[136]
transcription activator SNF2L2/SMARCA2	[137]
**“Stem Cell” Niche Invasion Angiogenesis**	
**Gene/Protein Name**	**Selected References**
HES-1	[138]
PEDF/EPC-1	[139]
cystine/glutamate transporter	[140]
zinc finger protein X-linked	[141]
selenium binding protein 1	[142]
CD24	[143,144]
SLIT2/Vasorin	[145]
**Signaling TGF-β Pathway?**	
**Gene/Protein Name**	**Selected References**
p21	[146]
NMA/BAMBI	[147]
MIC-1/Growth/differentiation factor 15	[148,149]
CDK-2 associated protein 2 (DOC 1R)	[150]
**Uncertain (Metabolism, Signaling?)**	
**Gene/Protein Name**	**Selected References**
transmembrane protein 50B/C21orf4	
TNFL9/41BBL/CD137	[151]
Sugen kinase 493/ hypothetical protein BC007901	
acetyl-coenzyme A synthetase 2-like	[152]

Genes/gene products (names in middle column) are functionally categorized as assorted in the left column, with relevant references shown in the right column.

Focusing on the 30 genes upregulated in DTT-treated U87 cells vs untreated U87 cells (Table 1), we further explored potential network/pathway relationships amongst the gene products via Ingenuity Pathway Analyses (IPA). Of the Top Networks revealed by the IPA algorithms, the 2 highest scoring Associated Network Functions were those of “Cell Death, Gene Expression, Free Radical Scavenging” and of “Gene Expression, Cellular Development, Cell Death” (interactomes shown in Figure 5D, E respectively). As noted in the names of the Top Networks, the relationships between cell proliferation and cell death via tumor cell manipulation of the UPR are evident here; in particular, the connections of these UPR-induced gene expressions and such important tumor signaling pathways as the PI3K/ERK, MAPK/AKT, VEGF/PDGF systems stand out (Figure 5D). In addition, the UPR-induced gene interactome interfaces with tumor-critical transcription factors such as FOS, HIFA, and SP1, as well as with members of the TGFβ family (Figure 5E). Table 2, consisting of categorizations of the genes via literature searches for function, emphasizes many of the same points.

**Figure 6:**
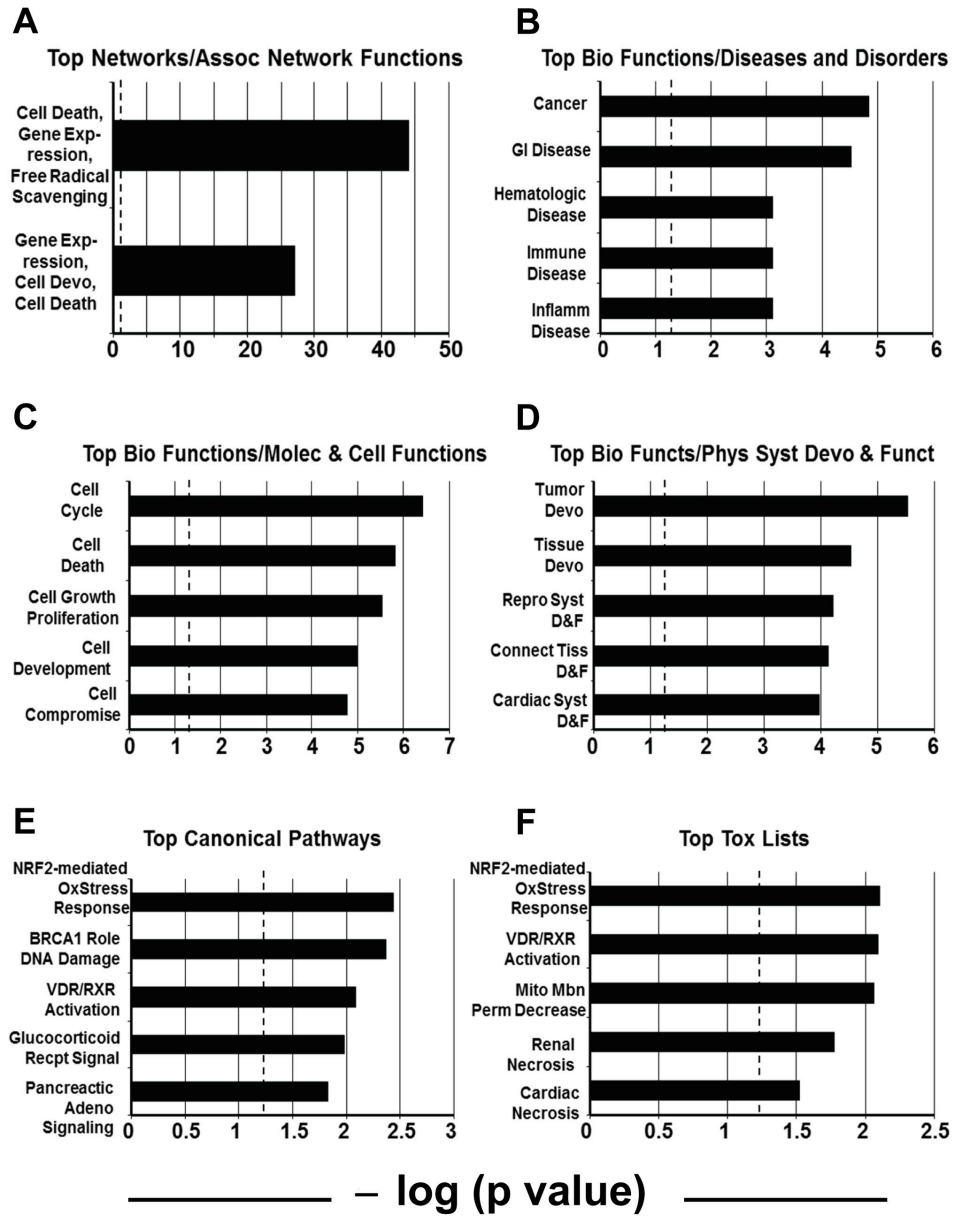
Integrated Pathway Core Analysis of the 30 upregulated polysome-fraction mRNAs (DTT-treated U87 cells vs untreated). Using Integrated Pathway Analysis algorithms, the identified messages were grouped into networks of associated functions, disease and toxicology relationships, and molecular, cellular, developmental, and physiological functions and pathways. Each grouping shows the top two or top five highest scoring categories (based on statistical significance from a Fisher Exact Test, significance set at p<0.05). X axes show this as a –log (p value), with hatched lines at 1.25 as the threshold for significance. Of the Top Networks, (**A**) shows the only significant Associated Network Functions identified for the 30 gene product set, “Cell Death, Gene Expression, Free Radical Scavenging” and “Gene Expression, Cellular Development, Cell Death”. (**B**, **C**, **D**) show the Top Biologic Functions with the following subheadings: In Diseases and Disorders (**B**), the top 5 categories were Cancer, Gastrointestinal Disease, Hematological Disease, Immunological Disease, and Inflammatory Disease. In Molecular and Cellular Functions (**C**), the top 5 categories are Cell Cycle, Cell Death, Cellular Growth and Proliferation, Cellular Development, and Cellular Compromise. In Physiological System Development and Function (**D**), the top 5 categories were Tumor Development, Tissue Development, Reproductive System Development and Function (“D & F”), Connective Tissue Development and Function, and Cardiovascular System Development and Function. There were 72 significantly scoring Biologic Functions overall (data not shown). (**E**) shows the top 5 (of 9 total) significantly scoring Canonical Pathways, including NRF-2 (Nuclear factor [erythroid-derived 2]-like 2)-mediated Oxidative Stress Response, Role of BRCA1 in DNA Damage Response, Vitamin D Receptor/Retinoic Acid X Receptor Activation, Glucocorticoid Receptor Signaling, and Pancreatic Adenocarcinoma Signaling. The Top Toxicology Lists (**F**) include NRF-2 (Nuclear factor [erythroid-derived 2]-like 2)-mediated Oxidative Stress Response, Vitamin D Receptor/Retinoic Acid X Receptor Activation, Decreased Permeability Transition of Mitochondria and Mitochondrial Membrane, Renal Necrosis/Cell Death, and Cardiac Necrosis/Cell Death.

A deeper systems analysis of the 30 upregulated mRNAs associated with polysomes following UPR induction in U87 cells puts a numeric value on the statistical significance of Figure 5D, E, (Figure 6A), showing that the Top Associated Network Functions for these genes had –log (p values) of 44 and 27, respectively, indicating that odds of these associations had occurred randomly were on the order of 10^44^ and 10^27^, respectively. Other high-scoring (highly significant) categories under Top Biologic Functions include “Cancer” under Diseases and Disorders (Figure 6B), “Cell Cycle”, “Cell Death”, and “Cell Growth and Proliferation” under Molecular and Cellular Functions (Figure 6C), and “Tumor Development” and “Tissue Development” under Physiological Systems Development and Function (Figure 6D). For both Top Canonical Pathways and Top Toxicology lists (Figure 6E, F, respectively) “NRF2-Mediated Oxidative Stress Response” and “Vitamin D/Retinoic Acid Receptor Activation” are significantly scoring categories. The PERK arm of the UPR can activate NRF2-mediated relief of oxidative stress during the UPR [54,55]; VDR and RXR have cross-talk in cells involving cell cycle and cell survival [56]. Thus, the 30 genes upregulated with acute UPR stress form integrated networks with known associations with cancer and cancer-related biologic properties.

### DTT-driven UPR induction leads to chemoresistance in U87MG cells treated with temozolomide

**Figure 7:**
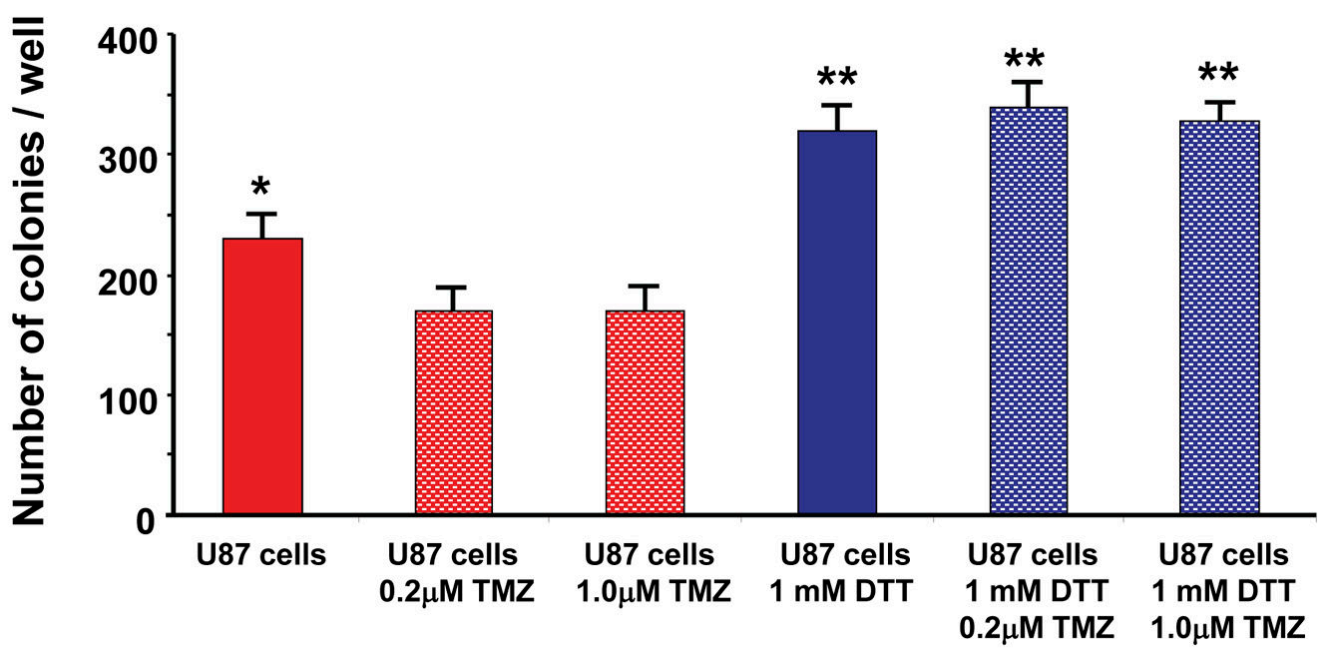
U87MG cells following UPR induction are resistant to temozolomide in clonogenic assays. U87 cells were either left untreated or stressed with 1 mM DTT (UPR inducer) for 4 hrs. Cells were then treated (or with vehicle control) with 0.2 or 1.0 µM temozolomide (TMZ) for 24 hrs. Cells were then washed and plated in soft agar for clonogenicity assays until a minimum of colonies of > 50 cells could be identified, which were then counted. Average colony counts with standard deviations are shown; ANOVA results of significant differences between groups (p < 0.05) are shown (drug-treated cells differed significantly in growth compared to untreated cells, which were also significantly different from DTT-treated cells, regardless of drug treatment).

The IPA and GO signatures for the networks of genes overexpressed upon UPR induction in U87 cells strongly suggested a role for chemoresistance in these cells. We tested this by performing clonogenic assays following UPR stress application to U87 cells with DTT, which were then treated with 200 or 1000 nM temozolomide (TMZ), the chemotherapeutic of choice in the standard of care for GBM treatment [1]. As shown in Figure 7, TMZ significantly reduces clonogenic colony outgrowth of the cells; UPR induction alone actually increases clonogenicity, and UPR induction prior to chemotherapy treatment completely abrogates the effects of the drug on U87 cells.

### Elevated levels of UPR transcription factors and ER chaperones correlate with poor patient prognosis

To extend our findings to human malignant gliomas, we analyzed a publicly available cDNA microarray database of 100 patient GBM cases for UPR induction patterns [57]. A key finding of that study [57] was the presence of three distinct gene subtypes of malignant gliomas that correlate patient survival with gene signatures: mesenchymal, proliferative and proneural. Tumors marked by either mesenchymal or proliferative gene signatures have significantly poorer prognoses than the proneural subgroups (61 weeks median survival vs. 171 weeks, respectively). In our analysis of this data set, we identified a 1.4- to 1.9- fold elevation (p<0.001) of BiP/GRP78, GRP94, and XBP-1 message levels in gliomas of the poorer prognostic subtypes (mesenchymal and proliferative) relative to the pronerual group (Table 3). This is within the range of other phenotypic markers that were linked to patient survival, including PTEN (1.57- fold increase in proneural vs. proliferative—loss of PTEN is common in high grade gliomas and generally indicative of shorter survival) and VEGF2 (1.6-fold increase in mesenchymal vs. proneural). Due to the wide-ranging heterogeneity of expression for certain genes of interest, the analysis was restricted to tumors with expression levels within three standard deviations from the mean. As seen in our analyses of glioma xenografts, CHOP mRNA (Figure 1A) and protein (Figure 3A,B) expression was variable, and may explain why elevated levels of this transcription factor were not statistically significant in this analysis. Parallel results were obtained when tumors were sorted by WHO grade, with elevated levels of XBP-1, BiP/GRP78 and GRP94 mRNA in grade IV GBMs (with necrosis) compared to the lower grade III tumors (Table 3).

**Table 3:** BiP/GRP78, GRP94, XBP-1, and CHOP/GADD153 expression in human high grade gliomas by diagnostic subtype (per Phillips et al, 2006) and by grade.

**Tumors by Diagnostic Subtype**									
	**BiP/GRP78**	**GRP94**	**XBP-1**	**CHOP**		**n**		**Age**	**Survival (wks)**
**Proneural^#^**	100%	100%	100%	100%		36		36.0	189.0
**Mesenchymal**	163%^**^	163%^**^	160%**	102%		31		48.5	87.0
**Proliferative**	191%**	165%**	142%**	124%		27		51.0	70.0
**Tumors by Histological Grade**									
	**BiP/GRP78**	**GRP94**	**XBP-1**	**CHOP**		**n**		**Age**	**Survival (wks)**
**WHO III^#^**	100%	100%	100%		100%		23	35.0	209.5
**IV (no necrosis)**	126%	130%	125%		102%		6	49.0	73.0
**IV (with necrosis)**	143%**	165%**	145%**		109%		68	48.0	85

^#^ Expression levels set to 100%; comparisons of elevated

^**^ p < 0.001

Age and survival are median values

**Figure 8:**
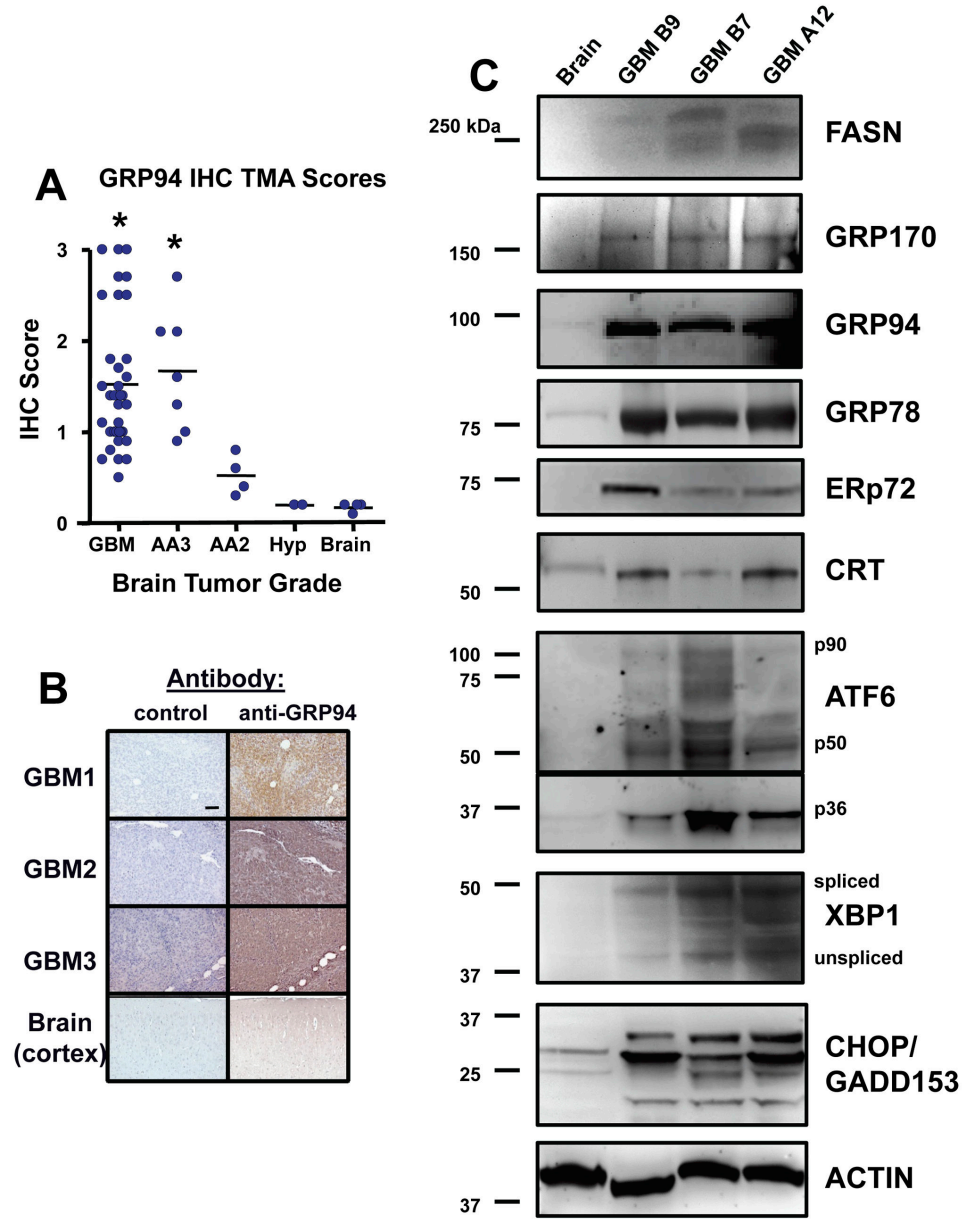
Immunohistochemistry and Western blots of patient brain tumors reveal high expression of UPR-related proteins. A Cybrdi “brain glioblastoma” tissue microarray (TMA) was probed for GRP94 by immunohistochemistry (IHC); (**A**) Scores were derived as describe in Materials and Methods, and show that high grade tumors such as glioblastoma multiforme (GBM, WHO grade IV) and anaplastic astrocytomas (WHO grade III—AA3) express significantly higher levels of GRP94 than do lower grade tumors (grade II anaplastic astrocytomas, AA2), anaplastic hyperplasia (Hyp) or normal brain (*, p < 0.05 by ANOVA comparing high grade gliomas vs the rest of the samples). Examples of the IHC staining are shown for 3 GBMs and normal brain (**B**). (**C**) Grade IV (GBM) tumor lysates (3 different tumors, not the same as those in **B**) and normal brain (cortex) lysates were separated by SDS-PAGE and electroblotted for Western blotting. Blots were probed with the antibodies listed as in Figures 2 and 3; “FASN” = Fatty Acid Synthase; “p90/p50/p36” = full length and cleaved forms of ATF6 “spliced/unspliced” = spliced or unspliced protein product of XBP1. Molecular weight markers are listed at left. Actin blot shown as loading control was a replicate for GRP78 and CRT.

Since GRP94 message in particular was highly upregulated in tumor types with poor prognoses, we extended these analyses by performing immunohistochemistry for GRP94 on a tissue microarray. This array contained over 30 high grade gliomas along with other lower grade tumors and benign growths. Immunohistochemistry scores for GRP94 were significantly higher in high grade gliomas (Grades III-IV/GBM) compared with lower grade tumors, astrocytic hyperplasia, or normal brain (Figure 8A, B). These findings confirm activation of the UPR pathway in human malignant glioma samples and suggest that ER-resident chaperones, in particular GRP94, and the UPR marker XBP-1 are indicative of highly aggressive forms of gliomas. These proteins may be useful prognostic indicators and suggest areas for drug targets aimed at UPR processes. For additional validation, we performed Western blot analyses on 3 patient GBM samples (compared to normal human frontal cortex) for fatty acid synthase (since lipid biosynthesis is a downstream activity of the UPR), for ER chaperones GRP170/ORP150, GRP94, GRP78, ERp72, and calreticulin, as well as the activated (spliced) and quiescent (unspliced) form of XBP-1. We included probes for the UPR transducer ATF6 and the downstream effector CHOP/GADD153 as well. The tumors universally displayed much higher expression of those proteins over normal brain (Figure 8C). Blots probed with actin antibodies as loading controls are shown in Supplemental Figure S6.

### The UPR elevates almost all levels of metabolism in stressed cells

**Figure 9:**
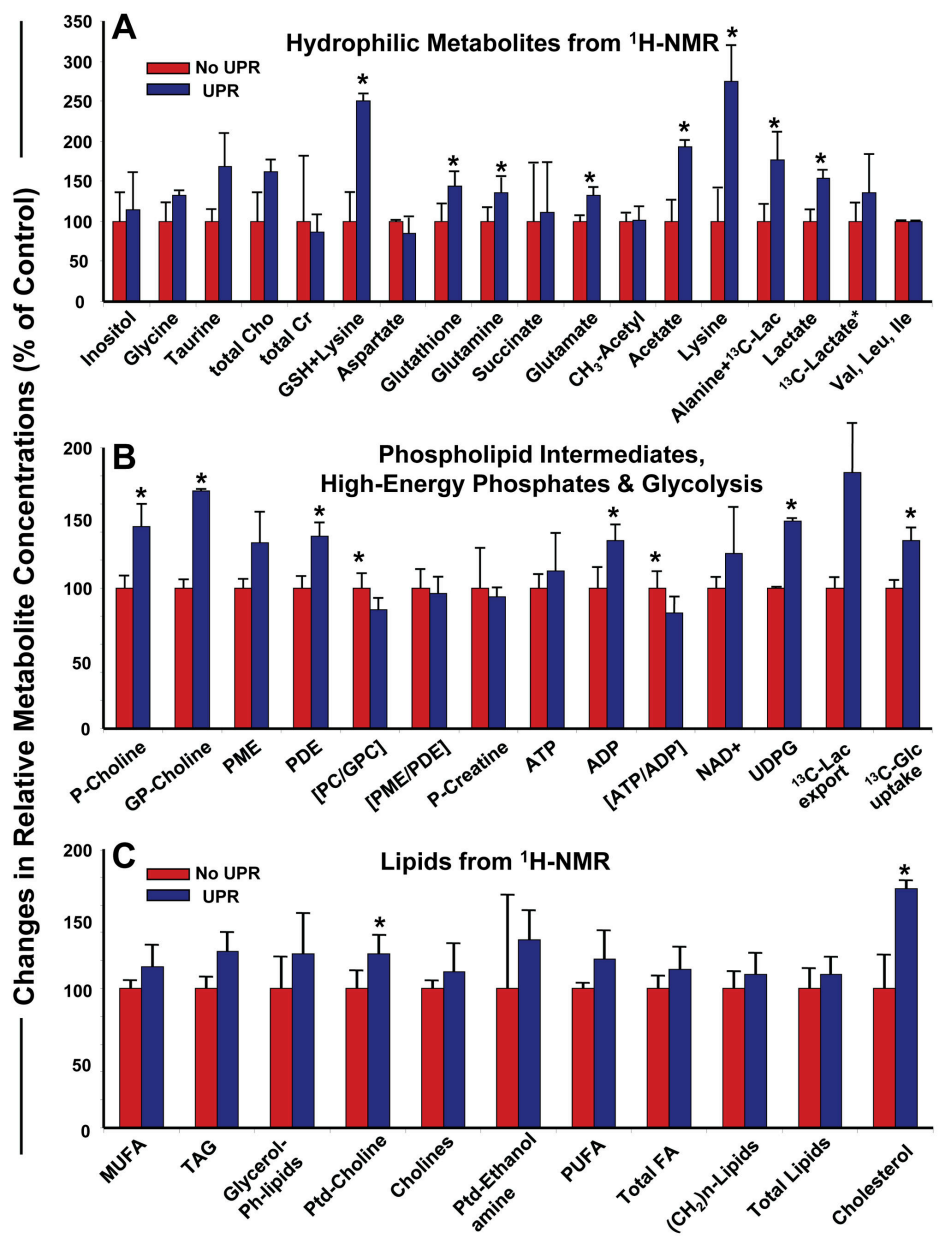
Relative metabolomic outputs of U87MG cells subjected to UPR stress compared to unstressed cells. U87 cells were grown in Knockout DMEM medium with serum replacement as described above. Cells were harvested, washed, and replated in the same (fresh) medium with or without 1 mM DTT, and with 5 mM ^13^C-glucose, for 4 hrs prior to cell and media harvest and PCA extraction as described in Materials and Methods. ^1^H-, ^31^P-, and ^13^C-NMR spectra were obtained and quantified; data analyses were conducted as described. Graphs compare metabolite components from untreated cells (set to 100%) vs treated cells; error bars show standard deviation, and * = p< 0.05 derived from Student’s *t* test comparing treated to untreated in averages of 3 separate experiments. (**A**) displays data for soluble metabolites. Cho = choline; Cr = creatine GSH = glutathione; Lac = lactate. (**B**) displays data for high energy phosphates and [1-^13^C] glucose uptake. P-Choline = phosphocholine; GP-Choline = glycerophosphocholine; PME = phosphomonoesters; PDE = phosphodiesters; PC = phosphocholine; GPC = glycerophosphocholine; P-Creatine = phosphocreatine; UDPG = uridine diphosphoglucose; Glc = glucose; Lac = lactate. (**C**) shows data for lipid compounds. MUFA = monounsaturated fatty acids; TAG = triacylglycerols; Glycerol-Plipids = glycerol phospholipids; PtdCholine = phosphatidylcholine; PtdEthanolamine = phosphatidylethanolamine; PUFA = polyunsaturated fatty acids; FA = fatty acids.

While the UPR is linked to lipogenesis [23,25], the connections to other metabolic pathways and cycles is less clear. The GO and IPA analyses and literature scrutiny of the 30 upregulated genes found in UPR conditions implied that various metabolic pathways might be engaged upon UPR stress (Figures 5 and 6, Table 2, and data not shown). Using ^13^C-glucose for metabolic flux analysis, we applied ^13^C-NMR spectroscopy to follow carbon uptake and metabolism and ^1^H- and ^31^P-NMR to quantify soluble and lipid metabolites/ phosphometabolites in U87MG cells following UPR induction with 1 mM DTT (4 hrs, ^13^C-glucose included at the same time; levels are normalized to the essential (branch-chained) amino acids valine, leucine, and isoleucine). Measures shown in Figure 9 are percentages compared to untreated U87 cells. Figure 9A shows that the UPR increases intracellular concentrations of many of the soluble metabolites measured, such as amino acids lysine, glutamine and glutamate (necessary for protein synthesis), acetate (increased ketone body/ lipid synthesis), and glutathione (increased oxidative defense). Most importantly, the UPR led to increased uptake of ^13^C-glucose and its fluxes through glycolysis (increased levels of lactate, alanine as well as UDPG, Figure 9A and B), two prominent metabolic “hallmarks” of tumor aggressiveness. As suggested above, relative lipid quantities are increased in UPR-induced cells (Figure 9C emphasizes ^1^H-NMR analysis on lipid extracts), with high statistical significance for increased cholesterol levels. Finally, strong evidence for elevated membrane turnover was seen in DTT stimulated U87MG cells. Increased levels of phosphatidylcholine (Ptd-choline, the major phospholipid in mammalian cell membrane, Figure 9C) and its metabolic intermediates phosphocholine and glycerophosphocholine (P-and GP-choline, Figure 9B) were evident in DTT treated cells. Previously, increased phospholipid synthesis (in particular, phosphocholine), and exacerbated glycolysis have been predictive of drug resistant clonal cell lines [58], which appears borne out in this glioma model as well (eg, TMZ resistance amidst UPR stress, Figure 7).

### Recurrent gliomas show increased lipogenesis compared to primary gliomas, along with selective UPR pathway activation

**Figure 10:**
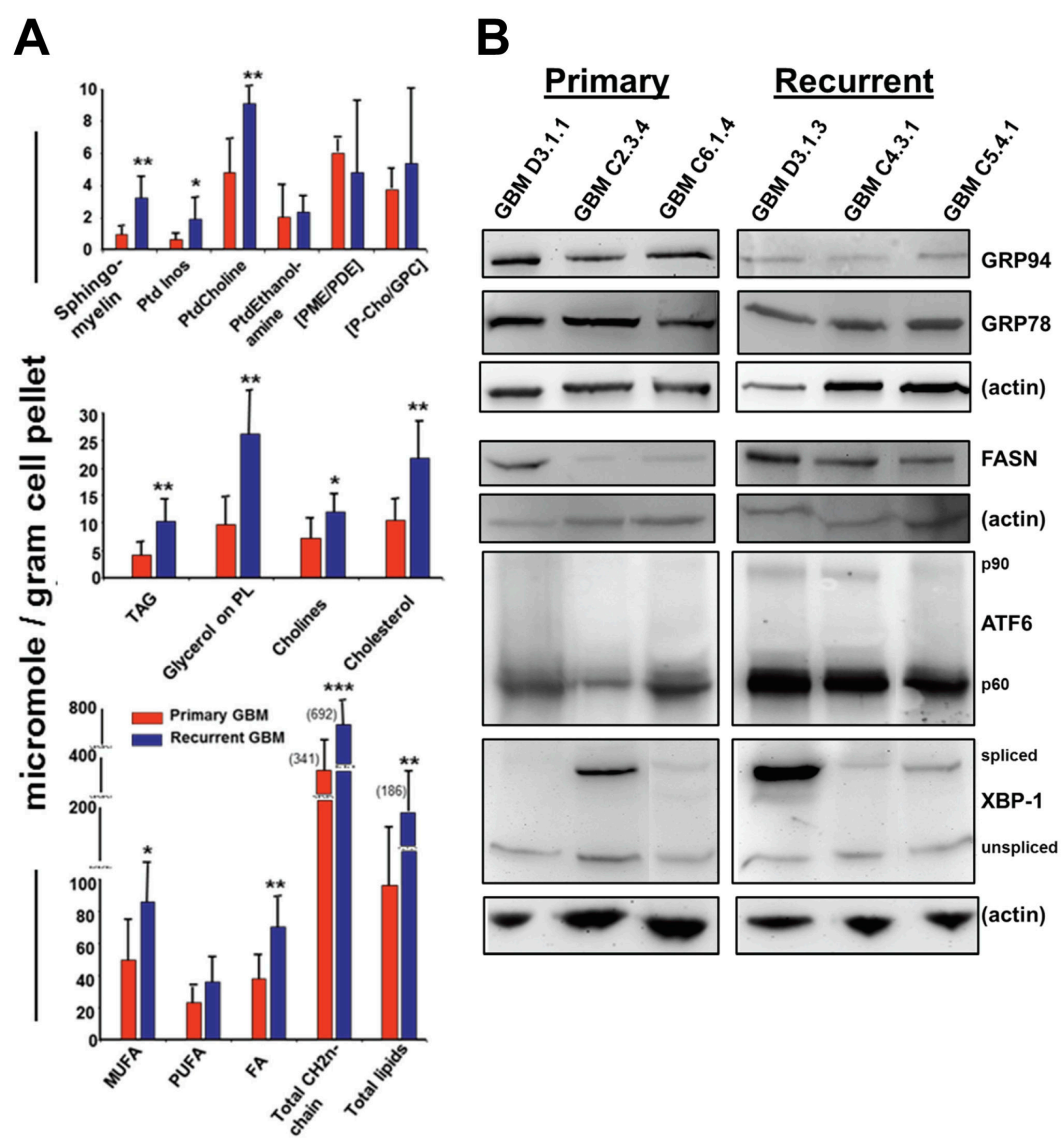
Recurrent gliomas show increased lipogenesis compared to primary gliomas, along with selective UPR pathway activation. Four primary and six recurrent GBM frozen samples were randomly selected from tumor tissues archived in our Tumor Bank. Tumors were extracted as described; ^1^H- and ^31^P-NMR spectra were obtained and quantified with data analyses conducted as described. (**A**) Graphs compare metabolite components (as micromoles per gram of starting tissue material) from the averages of the primary tumors (red bars) to the average values obtained from the recurrent tumors (blue bars). Error bars show standard deviation, and * = p< 0.05; ** = p< 0.01; *** = p< 0.001 derived from Student’s *t* test comparing primary tumor quantities to recurrent tumor quantities. Ptd Inos = phosphatidyl inositol; PtdCholine = phosphatidylcholine; PtdEthanolamine = phosphatidylethanolamine; PME = phosphomonoesters; PDE = phosphodiesters; P-Chol = phosphocholine; GPC = glycerophosphocholine; TAG = triacylglycerols; PL = phospholipids; MUFA = monounsaturated fatty acids; PUFA = polyunsaturated fatty acids; FA = fatty acids. (**B**) Western blots of primary and recurrent tumor lysates (the samples for which there were sufficient remaining materials to perform the immunoblot analyses) probed with antibodies against UPR components GRP94, GRP78, ATF6, and XBP-1, as well as fatty acid synthase (FASN). Actin blots are shown as loading controls.

Patients with GBMs generally undergo maximum surgical resection followed by chemoradiation therapy [59]. However, the tumors inevitably recur, usually within a year of diagnosis; the recurrent tumors exhibit further enhanced resistance to therapeutic attempts [60], prompting us to compare the metabolic and UPR profiles of primary versus recurrent GBMs. We found that nearly all of the lipid classes were significantly higher in recurrent versus primary GBMs (Figure 10A), while the quantities of hydrophobic metabolites, phosphate compounds, and glycolytic pathway members did not differ significantly between primary and recurrent tumors (data not shown). By Western blot we examined the amounts of particular UPR components as correlative to lipogenesis. To our surprise, the chaperones GRP94 and GRP78 seemed relatively downregulated in the recurrent tumors (Figure 10B), but the transcription factors ATF6 (active form p60) and XBP-1 (active/spliced form) are more generally upregulated. Fatty acid synthase, linked to XBP-1 activity [61], is also at higher levels in the recurrent tumors. Thus, the IRE1 and ATF6 pathways may be further activated in recurrent GBMs compared to primary tumors, with increased lipid production as a plausible outcome.

## Discussion

The objectives of this study were i) to perform a detailed and comprehensive molecular characterization of the unfolded protein response (UPR) in human glioma model tumors and tumor cells with the goal of establishing the presence of an active and/or constitutive UPR in these tumors (Figures 1-5, 8); ii) to determine dynamic and kinetic characteristics of the UPR upon induction in glioma cell lines (Figure 4), including the differential gene expression (Figures 5 and 6; Tables 1 and 2); iii) to determine if the UPR contributes to cell proliferation and chemoresistance in a glioma cell line (Figure 7); iv) to identify elements of the UPR in patient high grade gliomas (Figure 8) and correlate these to clinical outcome (Table 3); and v) to evaluate metabolic activation by the UPR in glioma cells (Table 2, Figure 9). This manifestation of the UPR in gliomas provides the tumors with all the benefits of the stress response--surviving or even thriving amidst the hostile environment--while simultaneously resisting UPR-driven apoptosis, and thus avoiding the potential costs of the UPR. A robust UPR would clearly benefit migratory or invasive tumor cells with surface and extracellular matrix remodeling capabilities, and with enhanced chaperoning of nascent proteins entering the secretory pathway. Coupled with additional high chaperone potential in the cytosol and other organelles due to increased expression of other chaperones/heat shock proteins [30], gliomas appear capable of prodigious protein expression necessary for their high proliferation rates as well. Metabolic remodeling emphasized in increased glycolysis, lipogenesis, membrane synthesis and oxidative stress defense would help the cells to survive in stress conditions while retaining their energy state and high proliferation rates. The limitations facing these cells *in situ* would thus be physical barriers, immune responses, and energy metabolism.

Our initial approach was to examine the levels of message and protein expression of UPR activators, effectors, and ER chaperones/residents in brain tumor cell lines and solid xenograft tumors, which we extended to polyribosome mRNA expression profiles of these samples (Figures 1-3, 5). These analyses revealed high levels of mRNA and proteins for most all of those UPR actors, as well as other members of the ER cohort. This was particularly evident in the solid tumors, where hypoxia, metabolic and environmental stress could lead to an essentially “chronic” UPR *in situ*. Established glioma cell lines (and to some extent, primary tumor cultures), however, did not seem to have a constitutive UPR, but instead displayed a highly inducible UPR through multiple classic pharmacologic stimulators, even compared to another tumor cell line. A notable feature of this activation was very transient decrease in overall protein synthesis and increased phosphorylation of eIF2α, which rapidly resolved into recovery of global and specific protein synthesis even amidst continued UPR stress (Figure 4). Thus, there is a prominent UPR activation profile that provides glioma cells/tumors with expanded ER protein-folding machinery and indications of increased translocon capacity (eg, high expression of TRAPα and Sec61α, Figure 1—Sec61α is considered a proto-oncogene responsive to ER stress [62]). This activation profile is readily inducible, and rather than leading to cell death despite prolonged stress, actually enhances glioma cell proliferation while providing potent resistance to chemotherapy agents (Figure 7). We also saw a shift in particular mRNA recruitment to polysomes upon DTT treatment of U87+EGFR cells that demonstrated a sedimentation profile similar to that of solid tumor-derived polysomes (Figure 5 and Supplemental Figure S5). It is not obvious at face value whether the UPR is prone to early activation in pre-cancerous astrocytes during tumorigenesis, or if intratumoral conditions drive UPR activation which later becomes essentially “fixed” in these tumors with the appearance of a “chronic” stressed state; our UPR activation data support the latter hypothesis.

The direct human relevance of these molecules and processes to brain tumors can be seen in our analysis of gene expression studies utilizing a publicly available cDNA microarray database from patients with high grade gliomas [57]. From that database three distinct gene signatures were defined and correlated with survival: mesenchymal, proliferative and proneural. Patients with tumors characterized in that publication as mesenchymal or proliferative suffered relatively poor median survival (61 weeks vs 171 weeks for proneural signatures), and we noted significant (p<0.001) elevation of GRP78/BiP, GRP94, and XBP-1 message levels in those poorer prognostic subtypes (Table 3). If the gene expression data were sorted by WHO grade, again the levels of GRP78/BiP, GRP94, and XBP-1 were significantly elevated in grade IV gliomas (GBMs, with necrosis) compared to the lower grade III tumors. We also saw similar elevated levels of UPR proteins, ER chaperones in particular, in Western blots of human GBM tissues (Figure 8). We verified high expression of GRP94 with immunohistochemistry on a tissue microarray of patient brain tumors where we found significantly higher levels of the ER chaperone in high grade gliomas compared to lower grade astrocytomas, CNS neoplasms, or normal brain. These results suggest that GRP94 may be useful as a biomarker or possibly a therapeutic target, in similar regard to HSP90 [63,64].

Along with relatively overexpressed ER residents and chaperones, we also noted high levels of UPR activators and effectors (Figures 2, 8) Among those effectors is ATF4, which has been identified by others as a highly expressed transcription factor in patient gliomas compared to normal brain [65]. ATF4 can trans-activate GADD34, resulting in dephosphorylation of eIF2α [66], which releases the translational block imposed by phospho-eIF2α, and global protein synthesis ensues. However, ATF4 also drives CHOP/GADD153 expression resulting in engagement of apoptotic signaling pathways, but since glioma cells are remarkably resistant to apoptosis [67], this arm of the UPR seems to have minimal effect in these tumor cells despite clear expression of CHOP (although it is somewhat variable, Figures 1, 2 and 8, Table 3). In the context of an actively growing tumor, the existence of high CHOP levels may reflect the tumor’s ability to tolerate the induction of pro-apoptotic pathways, and/or illustrate a heterogeneous response to stress throughout the tumor, or perhaps even subcellular localization differences between the cytosol and nucleus. In a model of tumor progression it is hypothesized that regional differences in hypoxia and nutrient diffusion may generate a series of metabolic niches within a growing glioma [68]. This may also involve UPR effectors such as ATF4 [13]. It is conceivable that distinct arms of the UPR pathway might also operate in a zone-specific manner in conjunction with areas of rapid proliferation, invasion, and apoptosis/necrosis. Indeed, high grade gliomas commonly have zones of differential invasiveness and proliferation [69], and a “necrotic core” [70,71] that may result from separation of these distinct UPR arms. This may be similar to the selective activation of discrete UPR components utilized in other biological scenarios, including the differentiation of plasma cells, which activates the IRE1/XBP-1 pathway to increase the secretory protein capacity of the cell, while inhibiting other UPR activities, including protein synthesis attenuation (PERK) and potentially destructive apoptotic cascades (CHOP) [72].

The 30 highly upregulated genes represented by mRNAs recruited to polyribosomes during UPR induction in DTT-treated cells (Table 1) may offer important insights into the processes that are significant to tumor cells utilizing such stress pathways. We chose polysome analysis because those mRNAs are on the verge of coding proteins and are thus likely to avoid the “translation gap” [73]. We have (cross) categorized the gene products into relevant functions relating to the UPR in Table 2, and have distinguished their functions and interactions with gene ontology and pathway analyses (Figures 5 and 6). These genes may be indicative of the initiation of a stress-response program in glioma cells that eventually becomes a chronic phenomenon resulting in the larger overall translational differential seen between *in vivo* grown tumors and *in vitro* stressed tumor cells (Figure 5). IPA interactomes of the upregulated gene products show key interactions with important tumor kinase and signaling systems including MAPK/AKT, PI3K/ERK, VEGF/PDGF, and FOS, HIFA, and SP1 transcription factor families. In addition, the classifications were also suggestive of altered metabolism at multiple levels.

One manifestation of the UPR is in lipid biosynthesis, largely thru activators downstream of XBP-1 [74]. In addition, some of the 30 genes upregulated during UPR induction are related to lipid metabolism. MIC-1 and PEDF are considered adipokines [75,76] and the latter is a regulator of hepatocyte triglyceride release [77], and curiously, G0S2 inhibits triacyglycerol hydrolysis [78]. Acetyl CoA synthase is necessary for acetate-dependent lipid synthesis in liver cancer cells [79]; thioredoxin reductase 1 expression is enhanced by particular phospholipids of oxidized LDLs [80], and oxLDL also upregulates Tribbles3 and p21 expression [81,82]. CD24 is considered a “gate-keeper” for entry of pro-migratory proteins such as β-1 integrin into lipid rafts [83]. The adipokine adiponectin induces BCL-3 expression [84]. The cystine/glutamate transporter (xCT or SLC7A11) is critical for L-cystine influx into the cell--then reduced to cysteine for glutathione synthesis, redox metabolism, and energy balance [85], particularly in hypoxic gliomas [86]. GDF15/MIC-1 is a pleiotropic growth factor with possible roles in systemic metabolism (eg, appetite) [87], and thioredoxin reductase 1 is a selenoprotein involved in numerous redox pathways [88]. HMOX1 is upregulated upon mitochondrial dysfunction, a situation common in tumors [89]. Curiously, CAMKK1 may be related to glycolytic activation induced by human cytomegalovirus (HCMV) [90], and while controversial, HCMV expression is reported in gliomas [91]. Thus, the expression array analyses and GO categorization (Figures 5 and 6, Table 2) converge on these aspects of metabolism.

UPR-driven metabolic changes are indeed exhibited in the metabolomics profiles shown in Figure 9. The key metabolites/ metabolic fluxes of glucose, lipid, phospholipid, protein and antioxidative pathways are increased in output following UPR induction in U87MG cells compared to control counterparts, including measures of glycolytic activity (^13^C-glucose uptake, ^13^C-lactate export, UDPG), cholesterol, acetate, phosphatidylcholine, amino acids and glutathione. Phenotypically these metabolome profiles are related to high proliferation and to drug resistance in tumor cells [58,92]. The chemotherapeutic agent in the standard of care for treatment of high grade gliomas is Temodar™ (temozolomide, TMZ), and this drug’s cytotoxic and/or cytostatic effects were lost when U87 cells were UPR-induced prior to chemotherapy treatment (Figure 7). ABC transporters such as ABC-1 [93] and BCRP, MRPs 1 and 4 [94] are expressed by U87 cells, and energy-dependent drug efflux via these transporters could utilize the elevated levels of energy precursors produced during the UPR. Increased lipid biosynthesis could allow for greater membrane trafficking of such transporters as well, or could perhaps result in increased extracellular vesicle release (in the form of exosomes or microvesicles) which have been shown to package and efflux drugs from cancer cells [95,96]. The UPR and oxidative stress may have a yin/yang relationship [97], and brain tumors in general have elevated oxidative stress phenotypes [98] (also seen in our own analysis, Figure 6) likely exacerbated by TMZ treatment. From our RNA (over)expression data we also note that SLC7A11 protects against oxidative stress [99]; extrapolating the gene expression data into their interactomes (Figure 5) brings the numerous pro-cancer signaling pathways into view along with their relationships to chemoresistance. For instance, DNA repair mechanisms known to be important in resistance to the DNA alkylating effects of TMZ [100] may be enhanced by PI3K/AKT signaling [101], with a role as well for MEK/ERK signaling [102]. NFκB signaling is also related to TMZ resistance in melanoma [103]. Thus, there are numerous potential molecular players, pathways, and metabolic cascades that may contribute to the chemoresistant phenomena seen in UPR-induced tumor cells. In addition, the role of autophagy in cell death [104] versus cell protection [105,106] looms large in this scenario. These are areas of ongoing investigation.

Further implications of metabolic changes potentially related to the UPR are seen in recurrent gliomas, where numerous lipid subclasses exhibit elevated intracellular levels compared to primary tumors (Figure 10A). Elevated choline levels, including free choline, phosphatidylcholine, phosphocholine, and glycerophosphocholine, have been related to malignant transformation and chemoresistance [107], and these compounds are key in spectroscopic imagining (MRSI) in gliomas [108]. Overall, there is little information comparing primary to recurrent gliomas for lipid content [109], and no connections between these tumor profiles and the UPR have been made. There are relationships between activated elements of the UPR and lipogenesis [23], and in particular XBP-1 is known to play roles in lipogenesis [61] via fatty acid synthase expression, but also inducing protein disulfide Isomerase expression [110]. We did not find increased expression of PDI in our comparisons of primary and recurrent tumors (data not shown), but our results suggest that ATF6 may be involved (Figure 10B). Literature reports indicate that ATF6 has divergent roles on lipogenesis and adipogenesis [111–113], so further evaluation of ATF6 in gliomas should prove important. The increased lipid products in recurrent tumors may reflect changes in membrane dynamics for tumor growth and invasion, possibly through extracellular vesicle production to alter the tumor microenvironment.

Our results here implicate various aspects of the UPR in glioma cell biology: continued protein synthesis despite ongoing ER stress; avoidance of apoptosis despite apparent activation of that arm of the UPR; recruitment of specific transcripts to polysomes encoding proteins involved in stress response, cellular metabolism, and maintenance of the “stem cell” niche; UPR-driven protection of cells from drug treatment; exacerbated cellular metabolism. These data strongly suggest that the UPR and its connections to tumor cell metabolism should be considered as high priority processes for future therapeutic targets and for continued studies in basic tumor biology and therapeutic resistance of high grade gliomas.

## Materials and Methods

### Ethics Statement for human (patient) tumor collection

Glioblastoma samples were obtained from the Neurosurgery Brain Tumor Bank, consisting of surgically-resected tumors from patients at the University of Colorado Hospital with appropriate Institutional Review Board approval. Tumor samples that came from pathology-confirmed Grade IV (glioblastomas, GBMs) were randomly chosen for lysate preparations. All specimens were completely anonymized. The study was approved by the Colorado Combined Institutional Review Board (COMIRB), 95-100 and 11-0749, and samples were collected following informed written consent.

### Ethics Statement for animal studies

This study was carried out in accordance with the recommendations from the Duke University Institutional Animal Care and Use Committee (DU IACUC). The protocols were approved by DU IACUC (Protocol A 159-02-05).

### Reagents

[^35^S] Methionine/cysteine and γ-[^32^P] dCTP were from MP Biomedicals (Irvine, CA). Cycloheximide, dithiothreitol, thapsigargin, and tunicamycin were from Sigma-Aldrich (St. Louis, MO). RNase OUT and TRIzol reagent were from Invitrogen (Carlsbad, CA). T4 polynucleotide kinase was from New England Biolabs (Ipswich, MA). All deuterated compounds and [1-^13^C] glucose for NMR were obtained from Cambridge Isotopes Laboratories (Andover, MA).

### Cell culture and UPR induction

U87MG, a cell line derived from an adult malignant glioma is available from the American Type Culture Collection (ATCC, Manassas, VA), HTB-14. It is the parental cell line of U87+EGFR and U87+EGFRvIII, which were obtained from Dr Webster Cavenee (Ludwig Institute, San Diego, CA) [114,115]. U87+EGFRvIII cells are transfected with the EGFR in-frame deletion mutant removing 801 base pairs from exons 2-7. U87MG cells and the indicated derivative lines were cultured as described [45]. For metabolomics preparations, U87MG cells were cultured in Knockout DMEM medium supplemented with 10% Serum Replacement and 5 ng/ml basic FGF and EGF, and 2 mM L-glutamine (Invitrogen). HeLa cells were obtained from the ATCC. For UPR induction experiments, cells were treated with either 1 mM DTT, 500 nM thapsigargin, 2.5µg/ml tunicamycin or vehicle control for 4 hours at 37^o^C. At the conclusion of the experiment, cells were rinsed in PBS and harvested into TRIzol reagent (Invitrogen, Carlsbad, CA). Total RNA was isolated from the TRIzol extracts according to the manufacturer’s instructions. For Western blots, cells were lysed as described [30].

### Animals and xenografts

BALB/c nu/nu athymic mice were obtained from the Duke University Department of Laboratory Animal Research in-house colony and were housed in Duke’s Cancer Center Isolation Facility. Animals were maintained in standard conditions. All animal studies were conducted under approved DU IACUC protocol (A159-02-05). This study was carried out in strict accordance with the recommendations in the Guide for the Care and Use of Laboratory Animals of the National Institutes of Health. All surgeries were performed under ketamine/isoflurane anesthesia, and all efforts were made to minimize suffering. When necessary, animals were euthanized by carbon dioxide inhalation.

D245MG is a transplantable xenograft tumor derived from a human adult high-grade glioma [116]. Subcutaneous and intracranial tumor transplantation of D245MG and U87 tumors was performed as described [117]. Tumors were harvested aseptically, rinsed in sterile saline, and were either flash-frozen in liquid nitrogen or were transported on ice for immediate processing. For RNA harvest, frozen tumors were crushed under liquid nitrogen in a mortar and pestle, homogenized in TRIzol reagent and total RNA isolated according the manufacturer’s instructions. Total RNA samples derived from solid tumors were subjected to an additional round of TRIzol extraction prior to analysis.

### Human glioma samples

GBM samples were obtained from the Neurosurgery Brain Tumor Bank, consisting of surgically-resected tumors from patients at the University of Colorado Hospital with appropriate Institutional Review Board approval. Tumor samples that came from pathology-confirmed Grade IV (glioblastomas, GBMs) were randomly chosen for lysate or metabolomics preparations. Two tumors were dissociated into cell suspensions immediately after surgical resection and were treated as primary cultures for some of the experiments; these were used within 2 weeks of their resection and dissociation. Briefly, tumors were placed in Neurobasal A medium supplemented with 2 mM L-glutamine, 1x N2 and B27, and 5 ng/ml basic fibroblast growth factor and epidermal growth factor (all reagents from Invitrogen). The tumors were diced into small pieces with a scalpel, and remaining pieces were further disrupted with the flat portion of a 3cc syringe plunger. The cell-containing medium was passed through a 100 µm nylon mesh, and the cells were pelleted by centrifugation. Cells were resuspended, counted and placed at 1 million cells per ml in T75 flasks in 10 ml of the aforementioned Neurobasal A medium + supplements. Cultures were treated with UPR inducers and were collected for lysis and Western blotting as described above and below.

### Tumor/cell lysate preparation and Western blotting

Cell lysates were prepared as described above. Tumor lysates were prepared by sonicating diced tumor fragments in RIPA buffer (50 mM Tris-HCl/150 mM NaCl, pH 7.4; 1% NP-40; 0.25% sodium deoxycholate; 1 mM EDTA) with added protease inhibitors (Complete Protease Inhibitor Tablet, Roche, Indianapolis IN, per 25 ml of extraction buffer). Lysates were incubated on ice for 5 min, followed by centrifugation (10,000 x g, 15 min, 4^o^C). The supernatants were used in subsequent experiments. Protein concentrations were determined using a Bradford assay with serum albumin as standard. Twenty µg samples were prepared in SDS sample buffer with 2-mercaptoethanol and electrophoresed on a 4-12% gradient Bis-Tris gels (Invitrogen, Carslbad, CA) or on 10% Bio-Rad Criterion gels (Bio-Rad, Hercules, CA). The proteins were then transferred to nitrocellulose membranes using an iBlot system (Invitrogen). Membranes were blocked by incubation in 3% milk/PBS+ 0.5% Tween-20(PBST) overnight at 4° C. The blots were then probed with the following antibodies at a 1:1000 dilution in 1% milk/PBST (from Assay Designs, Ann Arbor, MI): anti-calnexin (SPA-860), anti-GRP 78 (SPA-826), anti-ERp72 (SPA-720), anti-PDI (SPA-890), anti-calreticulin (SPA-601), anti-GRP94 (SPA-850). From Abcam (Cambridge, MA) we used anti-ATF4 (ab1371) and anti-CHOP/GADD153 (ab53081), dilutions of 1:1000. From Cell Signaling (Boston, MA), we used anti-eIF2α (9722), anti-phospho- eIF2α (9721), and anti-fatty acid synthase (3189) at 1:100 dilutions. Other antibodies included anti-ORP150/GRP170 (Immuno-Biological Laboratories, Gunma, Japan) used at 1:1000 dilution, anti-HERP (Biomol International, Plymouth Meeting, PA) at a 1:2000 dilution, anti-XBP-1 (sc7160, Santa Cruz Biotechnology, Santa Cruz, CA) used at 1:1000 dilution, anti-ATF6 (MAB0082, Abnova, Taipei, Taiwan) used at a 1:1000 dilution, and an anti-TRAPα antibody (CVN, Duke University) used at 1:2000. Rabbit antibody to Sec61α was also from CVN; for Sec61α blots, SDS-PAGE gels were transferred in 10mM CAPS buffer (pH 10.5). Antibody dilution was 1:5000. Anti-β-actin antibody (A5316, Sigma-Aldrich, St Louis, MO) was used as a control; blots were stripped and re-probed as necessary, or replicate blots—those loaded with the same amounts/volumes as the indicated blots but were probed separately for actin--were probed as indicated in the Figure Legends and Supplemental Figure Legends. Appropriate species-specific secondary antibodies (anti-rat, anti-mouse, or anti-rabbit IgG-HRP conjugates) were used at 1:5000 dilutions (all from GE HealthSciences/Amersham, Piscataway, NJ, USA). Following secondary antibody incubations, membranes were washed in PBST, dH2O, and incubated in enhanced chemiluminescent substrate (Pierce Chemical Co., Rockford, IL) for 5 min prior to exposure to Kodak BioMax autoradiography film (Kodak, Rochester, NY) or to electronic imaging in a FluroChem Q Imager III device (ProteinSimple, Santa Clara CA). Blots were re-probed (or identical blots probed) with an anti-actin antibody (Sigma-Aldrich, St. Louis, MO) followed by secondary antibodies and development as described. Densitometry determinations were made using ImageJ (http://rsbweb.nih.gov/ij/).

### Immunohistochemistry

For immunohistochemistry, 5 µm-thick sections of formalin-fixed, paraffin-embedded tumor or murine brain were placed on positively charged slides. The slides were deparaffinized, and antigen retrieval was performed by microwave heating in 10mM citrate buffer (pH 6.0, Zymed Laboratories, South San Francisco, CA), or with proteinase K treatment (Dako USA, Carpinteria CA), followed by treatment with 0.3% H_2_O_2_ in methanol. After blocking in serum matched to the secondary antibodies for 1 hr, the sections were incubated with primary antibodies at 4^o^C overnight. Slides were washed in PBS, and incubated with biotinylated secondary antibodies at room temperature for one hour. This was followed by washing and addition of tertiary streptavidin HRP conjugate (Vectastain ABC kit, Vector Laboratories, Burlingame, CA) for 30 min at room temperature. Slides were washed, and the immunostain was developed with DAB solution (Pierce Chemical Co, Rockford, IL) for 5 min. After counterstaining, the sections were mounted with cover slips for observation. The antibodies used were the same as those used in Western blotting, except that a rabbit anti-GRP94 antibody (DU-120, developed by Dr Chris Nicchitta, Duke University) was used, as was anti-IRE-1 (sc20790, H-160, Santa Cruz) Antibody dilutions/concentrations used: anti-ERp72, 1:10,000; anti-ORP150/GRO170, 1 µg/ml; anti-GRP94, 0.5 µg/ml; anti-calnexin, 1:4000; anti-GRP78, 2 µg/ml; anti-ERp72, 1:10,000; anti-protein disulfide isomerase, 0.5 µg/ml; anti-HERP, 1:10,000; anti-calreticulin, 1:4000; anti-CHOP, 0.25 µg/ml; anti-XPB-1, 0.1 µg/ml; anti-IRE1, 0.5 µg/ml; anti ATF4, 0.1 µg/ml; normal rabbit IgG, normal rabbit serum, or isotype-matched mouse IgG were used as negative controls at the same concentration or dilution as the primary antibodies. Secondary antibodies were a biotinylated goat anti-rabbit IgG or goat anti-mouse IgG used at 1:200 dilution (Zymed).

For assessment of GRP94 immunoreactivity on human patient brain tumor samples, a Cybrdi “brain glioblastoma” tissue microarray (TMA, CS17-01, Cybrdi Inc, Frederick, MD) was screened. This array includes high grade gliomas (GBMs/anaplastic astrocytomas grade IV, and grade III) as well as lower grade tumors (grade II, astrocytic hyperplasia) and normal brain specimens. Arrays consisted of formalin-fixed 5 µm-thick sections with dots of 1.5 mm, spotted in duplicate for each tissue. Staining was scored by an intensity scale of 0 (no stain) to 4 (highest) times the fraction of cells staining. Scores for individual tumor spots differed from each other by less than 10%, and were averaged for individual tumor sections. Lowest possible score is 0.0, highest is 4.0.

### Northern Blot Analysis

Northern blots protocols have been described [118]. Briefly, RNA was resolved on 3% formaldehyde 1% agarose gels, and transferred in 5X SSC, 10 mM NaOH by downward capillary flow onto Hybond membrane for 2 h. Probes were generated from oligonucleotide DNA primers and included the following:

GRP94: (CCTCTACTGCTTCATCATCAGATTCTTCTTTCTCTTCT);
GRP78/BiP: (GTCTTCCTCAGCAAACTTGTCAGCATCATTAACCATCCCCAGTTCCCG);
XBP-1: (TCAGGTCGCTGAGGCGCTGTCGCTTGC);
CHOP (TCTCTTCAGCTAGCTGTGCCACTTTCC);
ATF4: (CCTAGGCTTTCTTCAGCCCCCAAACCCGAC);
ATF6: (AGGTTTAGTCACGGAAAGTTTTCCATTCAC);
GAPDH: (GGGGCCATCCACAGTCTTCTGGGTGGCACTGATGGCATGG).

Probes were end-labeled with [γ-^32^P] dCTP using T4 polynucleotide kinase. PhosphorImager plates were scanned using a Typhoon 9400 (GE Healthcare, Piscataway, NJ), and data analyses were performed using the ImageQuantTL software (GE Healthcare).

### Protein Synthesis Assays

Cells were treated with 1mM DTT for the indicated times and subsequently radiolabeled by the addition of 100 µCi/ml of [^35^S] methionine to cell culture media (minus methionine) suspensions. Cells were lysed in 1 ml of 1% NP-40, 0.05% SDS, 25 mM Tris, pH 7.5, 150 mM NaCl, 5 mM EDTA, 1 mM DTT, and 1 mM PMSF for 10 min on ice and clarified by centrifugation. For levels of newly-synthesized total protein, trichloroacetic acid (TCA)-precipitated lysates were collected onto Whatman paper and radiolabel incorporation was determined by scintillation counting of the tricholoacetic acid/ethanol: ether treated filter squares [119]. Immunoprecipitation of radiolabeled proteins was performed using the indicated antibodies and protein A-Sepharose resin as described previously [119].

### Velocity Sedimentation

Freshly isolated tumors were minced, homogenized in chilled buffer (1% Triton X-100, 1% DOC, 400 mM KOAc, 25mM KHepes, 15mM MgOAc, 1mM DTT, 200µM cycloheximide, 80U/ml RNAse Out), and centrifuged to remove debris (15 min, 10,000 x g). The resulting supernatant fraction was loaded onto 10 ml 15–50% linear sucrose gradients and centrifuged at 151,000 x g as described previously [120]. Fractions were collected with an ISCO gradient collector (Teledyne/Isco, Lincoln, NE), with continuous A_254_ nm recording. For northern blot analysis, RNA was isolated from individual gradient fractions by phenol-chloroform extraction.

### Microarray Methods and Analysis

Triplicate extracts of either control U87 tissue culture cells, acutely stressed U87+EGFR tissue culture cells (1mM DTT, 4h) or U87 solid tumor xenografts were subjected to velocity sedimentation, as described above, and total RNA fractions isolated from the polyribosome region (defined as disome to terminal polyribosome) for each individual gradient. All microarray processing and analysis was completed at the Duke University Microarray Facility. Detailed processing and analysis protocols can be found at the Duke Microarray Facility Website at http://microarray.genome.duke.edu. Briefly, RNA quality was assessed by UV spectroscopy and electrophoresis on an Agilent Lab-on-a-Chip 2100 Bioanalyzer (Agilent Technologies, Palo Alto, CA) in accordance with the manufacturer’s recommendations. Total RNA from each sample and the reference (Universal Human Reference RNA, Stratagene, La Jolla, CA) was hybridized to oligo(dT) primers at 65° C. Subsequently, the mixture was incubated 42° C for 2 hours with the addition of reverse transcriptase (Superscript II, Invitrogen, Carlsbad, CA), Cy5- or Cy3-dUTP, Cy5- or Cy3-dCTP, and a dNTP mix. Sample and reference cDNA were pooled, mixed with 1x hybridization buffer (50% formamide, 5x SSC, and 0.1% SDS), COT-1 DNA, and polydeoxyadenylic acid to limit nonspecific binding, and heated to 95° C for 2 minutes. This mixture was pipetted onto the microarray slide (Operon Human Oligo Set, version 4.0; 35,035 probes) and hybridized overnight at 42° C. The array was then washed at increasing stringencies and scanned on a GenePix 4000B microarray scanner (Axon Instruments, Foster City, CA). Analyses were performed using GeneSpring software where intensity-dependent (Lowness) normalizations were applied on the entire data set. A one-way parametric analysis of variance (ANOVA) tests were performed with a *P* value of 0.01 (1085 genes). To identify genes that were differentially expressed between the acutely-stressed cell culture sample and the tumor samples, gene ontology (GO) analysis was performed with GeneSpring for groups enriched 2-fold or more in the tumor than either cell culture model, using all genes with 2 of 3 replicates containing a p<0.05 and where 5% or more of the total genes for each GO group are represented in the tumor. Ingenuity Pathway Analysis (IPA) (http://www.ingenuity.com/products/pathways_analysis.html) was also used to classify relationships between gene products with interactome design and development of functional, biologic, and toxicological associations.

### Methods and statistics for analysis of human GBM database

Previously published data [57] from 100 human patient samples were obtained from the Gene Expression Omnibus # GSE4271 (http://www.ncbi.nlm.nih.gov/geo/). The mRNA expression levels (denoted by the MAS5-calculated signal intensity) of UPR-induced transcripts (BiP/GRP78, GRP94, XBP1 and CHOP) were obtained for each of the 100 tumor samples and analyzed for statistical correlations between transcript expression levels and any of the following three analysis categories: tumor subgroup, tumor recurrence, or tumor grade. Values were grouped into each of the three categories, averages of mRNA expression for each UPR-transcript in the subgroup were calculated, and individual tumors with values within three standard deviations from the mean were included in the analysis. Percent enrichment was determined as a ratio of median values of the poorer prognostic subgroups (including WHO grade IV with and without necrosis; mesenchymal and proliferative) to the more benign subgroups, which was set at 100% (including WHO grade III and proneural). Median values of patient age and survival in weeks for each subgroup were also calculated. Statistical significance was evaluated by two-tailed Student’s *t* test.

### Clonogenic assays

U87MG cells were grown in Knockout DMEM with serum replacement and FGF/bFGF as described above. Cells were subjected to UPR induction (or not) with 1mM DTT for 4 hrs. Following that, cells were treated (or not) with 200 or 1000 nM temozolomide (TMZ, Sigma-Aldrich) or vehicle control for 24 hrs. Cells were then plated in a soft agar matrix in six-well plates at a density of 15,000 cells per well. Each treatment condition was plated in triplicate. Cells were fed every 48 hours and allowed to grow until both treated and untreated groups had colonies that contained ≥50 cells (day 8). Colonies at that time point were then stained with crystal violet and counted using a microscope.

### NMR-Based Metabolomics

Cell extraction for nuclear magnetic resonance (NMR) spectroscopy. For NMR experiments, cells were incubated with 5 mM [1-^13^C]-glucose for 4 h before perchloric acid (PCA) extraction (for stressed U87 cells, 1 mM DTT was added at the same time). All cell extractions were performed using a previously published perchloric acid extraction protocol allowing for water-soluble and lipid fraction separation [121]. Lyophilized media and water-soluble cell extracts were re-dissolved in 1.5 and 0.5 mL of deuterium oxide respectively. After centrifugation, the supernatants were neutralized to pH 7.2 to allow precise chemical shift assignments. Lipid extracts were re-dissolved in 1 mL mixture of deuterated methanol/chloroform (2:1, v/v). *NMR Spectroscopy*. High-resolution ^1^H- and ^13^C-NMR experiments were performed with the Bruker 500 MHz DRX spectrometer equipped with an inverse 5 mm TXI probe and ^31^P-NMR experiments with the 300 MHz Bruker Avance system with a 5 mm QNP probe. For proton NMR, a standard water presaturation pulse program was used for water suppression; spectra were obtained at 12 ppm spectral width, 32 K data arrays, 64 scans with 90 degree pulses applied every 12.8 s. Trimethylsilyl propionic-2,2,3,3, -d_4_ acid (TSP, 0.5 mM) was used as an external standard for metabolite chemical shift assignment (0 ppm) and quantification (for exact metabolite assignment and their chemical shifts refer to [122]. ^13^C-NMR spectra with proton decoupling were recorded using the C3-lactate peak at 21 ppm as chemical shift reference (spectral width was 150 ppm, 16 K data arrays, 20 K scans applied every 3 s). For quantification of absolute concentrations of ^13^C-metabolites, the possible positions for ^13^C-labelling of the metabolite of glycolysis or the tricarboxylic acid (TCA) cycle after incubation of cells with [1-^13^C]-glucose were determined as in [58]. [3-^13^C]-lactate satellite peak (at 1.23 ppm) from ^1^H-NMR spectra served as an internal standard for ^13^C-NMR spectra (at 21 ppm) for calculation of ^13^C-enrichment of glucose and glucose metabolites [121,123]. In order to confirm that ^13^C-NMR-based calculations of *de novo* [1-^13^C]-glucose uptake and metabolism are correct, we also performed standard enzymatic analysis on the same medium spectra (see below). ^31^P-NMR spectra were obtained using the spectral width of 50 ppm and 16 K data arrays, with 6–10 K scans being applied every 3.5 s. Before the ^31^P-NMR spectra were recorded, EDTA (100 mM) was added to each PCA extract to complex divalent cations. Methylene diphosphonic acid (2 mM) was used as an external standard for chemical shift references (18.6 ppm) and for metabolite quantification in ^31^P-NMR. All data were processed using the Bruker WINNMR programme. All NMR experiments were performed at the University of Colorado Cancer Center/ CTSA Metabolomics Core.

NMR-based metabolomics profiling of viable tumor tissue. GBM samples were flash-frozen in the operating room and stored frozen. Tumors were individually extracted for multinuclear NMR following published protocols [121]; weighed tumors were extracted with perchloric acid (PCA) to obtain aqueous and lipid phases. Lyophilized PCA extracts were redissolved in deuterium oxide, the lipid extracts in deuterated chloroform/ methanol. NMR experiments were performed in a 500MHz Bruker Avance NMR spectrometer with 5-mm TXI/ QNP probe heads. For proton NMR (fully-relaxed, water-suppressed), trimethyl propionic-2,2,3,3, -d4 acid (TSP, 0 ppm) (TSP, 0.5 mM for cell PCA extracts and 1.2 mM for media) was used as an external standard for metabolite quantification. Before ^31^P MR spectra were recorded, EDTA (100mM) was added to PCA extracts to complex divalent cations. Methylene diphosphoric acid (18.6 ppm; 3 mM) was used as an external standard for metabolite quantification in ^31^P NMR. The data were processed using the XWINNMR program and TopSpin software.

### Statistical analyses

Unless otherwise stated, where applicable results are presented as means +/- standard deviation (SD) for each series of experiments with Student’s *t*-test was used to determine significance of data; significance was set at *p* < 0.05. For other cases, one-way ANOVA was performed followed by Tukey’s post hoc multiple comparison tests, where p< 0.05 was chosen a significant unless otherwise stated (SPSS 20) (http://www-01.ibm.com/software/analytics/spss/?pgel=ibmhzn&cm_re=masthead-_-products-_-sw-sps). Statistics used for IPA can be found at the website http://www.ingenuity.com/index.html. All experiments were performed a minimum of two times.

## Supporting Information

Figure S1 **Replicate or stripped/reprobed blots from Figure 1E probed with an anti-actin antibody as a loading control.** Blots for GRPs 170 and 78, for ERp72 and TRAPα were replicate blots. Blots for GRP94, CNX, CRT, HERP, and Sec61α were stripped and reprobed for actin.
(TIF)

Figure S2 **Upregulation of ER resident protein expression in xenograft human gliomas seen in immunohistochemistry.** Representative immunohistochemical staining from paraffin-embedded, formaldehyde-fixed tissue sections of normal brain from *nu/nu* mice and xenografts from U87+EGFR, U8MG7, and D245MG samples. Control panels are probed with a species-matched irrelevant antibody at concentrations identical to the experimental/primary antibody. The scale bar represents 100 µm.
(TIF)

Figure S3 **Replicate or stripped/reprobed blots from Figure 3 probed with an anti-actin antibody as a loading control.** Blots for GRPs 170 and 78, for ERp72, and for CRT were stripped and re-probed with an actin antibody. Blots for GRP94 and HERP, for ATF6, and for XBP-1 are replicate blots.
(TIF)

Figure S4 **Treatment of glioma cell cultures with other chemical inducers upregulates UPR-related protein expression.** U87MG cells and the primary GBM culture model GBM-P9 were treated left untreated (“NoTx”) or were treated with tunicamycin (“Tuni”) or thapsigargin (“Thaps”) as described in Figure 4. Cells were harvested, lysed, and proteins separated by SDS-PAGE, followed by transfer to nitrocellulose for probing in Western blots with the antibodies listed (and their respective actin loading controls). Blotsfor GRP94, GRP78, and ERp72 were stripped and re-probed with actin antibodies. Blots forCRT, CHOP, HERP, and XBP-1 are replicates probed with actin antibodies.
(TIF)

Figure S5 **Xenograft tumors exhibit steady-state polyribosome loading of UPR-response transcripts.** Polyribosomes were obtained from normal murine brain and solid tumors of both the U87MG and U87+EGFR glioma models. Following homogenization, sample lysates were layered over a linear sucrose gradient (15-50%), separated at 150,000x g for 3 hours, and the gradients fractionated with an automated gradient fractionator, with continuous UV (254 nm) absorbance monitoring. Downward-pointing arrows indicate sedimentation of 80S monosomes. RNA was extracted from individual gradient fractions and analyzed via Northern blot for ATF4, GRP94, BiP/GRP78 and GAPDH mRNA content.
(TIF)

Figure S6 **Replicate or stripped/reprobed blots from Figure 8C probed with an anti-actin antibody as a loading control.** Blots for FASN and ERp72, for GRP170 and CHOP, for ATF6, for XBP-1, and for GRP78 and CRT, are replicate blots. Blots for GRP94 were stripped and re-probed for actin.
(TIF)
