# Supplementary figures and images for: Induction of the Unfolded Protein Response Drives Enhanced Metabolism and Chemoresistance in Glioma Cells
Source: PLoS One. 2013 Aug 15;8(8):e73267. doi: 10.1371/journal.pone.0073267 (PMC3748289; doi:10.1371/journal.pone.0073267)

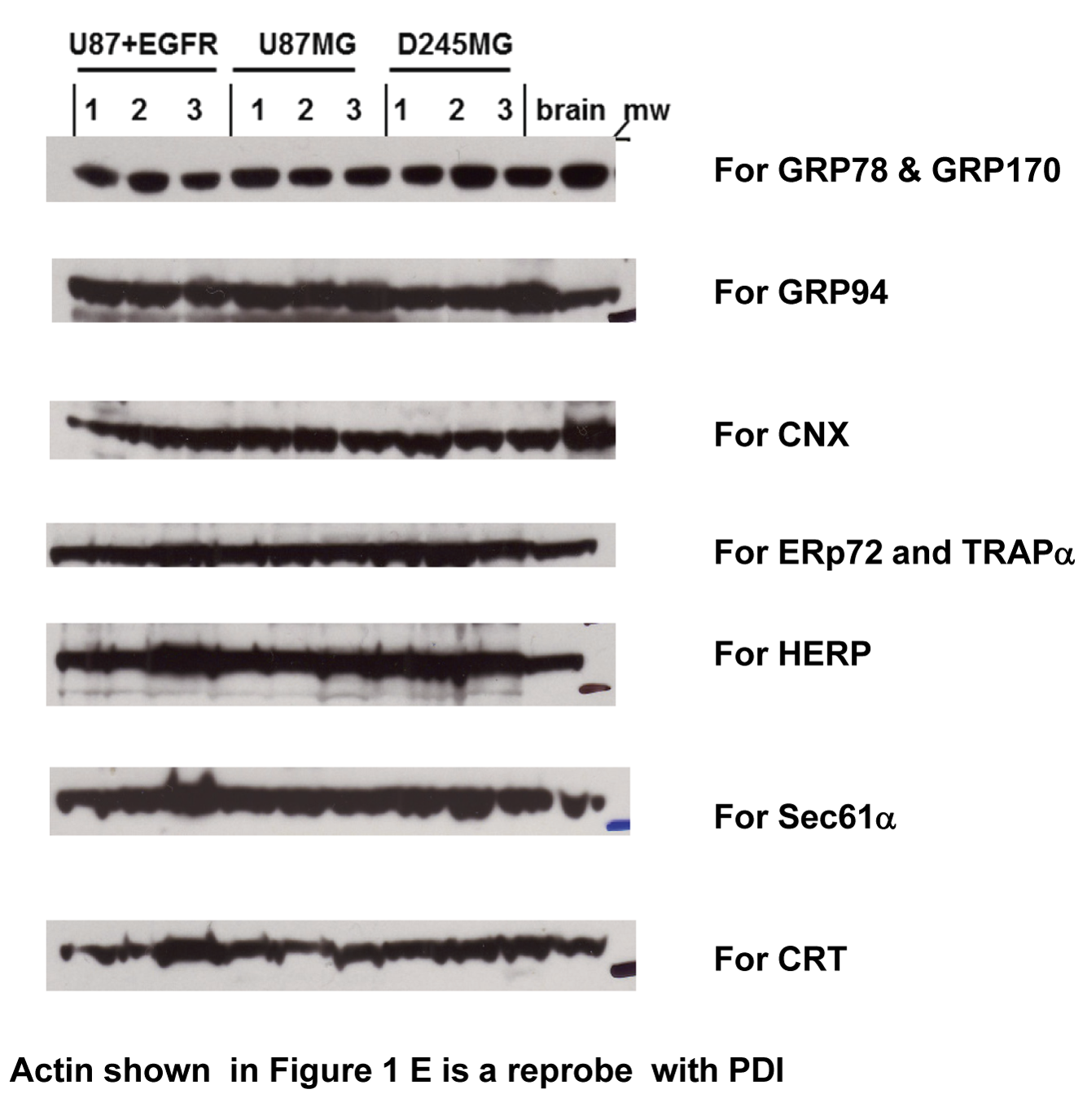

Supplement: Figure S1 — Replicate or stripped/reprobed blots from Figure 1E probed with an anti-actin antibody as a loading control. Blots for GRPs 170 and 78, for ERp72 and TRAPα were replicate blots. Blots for GRP94, CNX, CRT, HERP, and Sec61α were stripped and reprobed for actin. (TIF) [file pone.0073267.s001.tif]

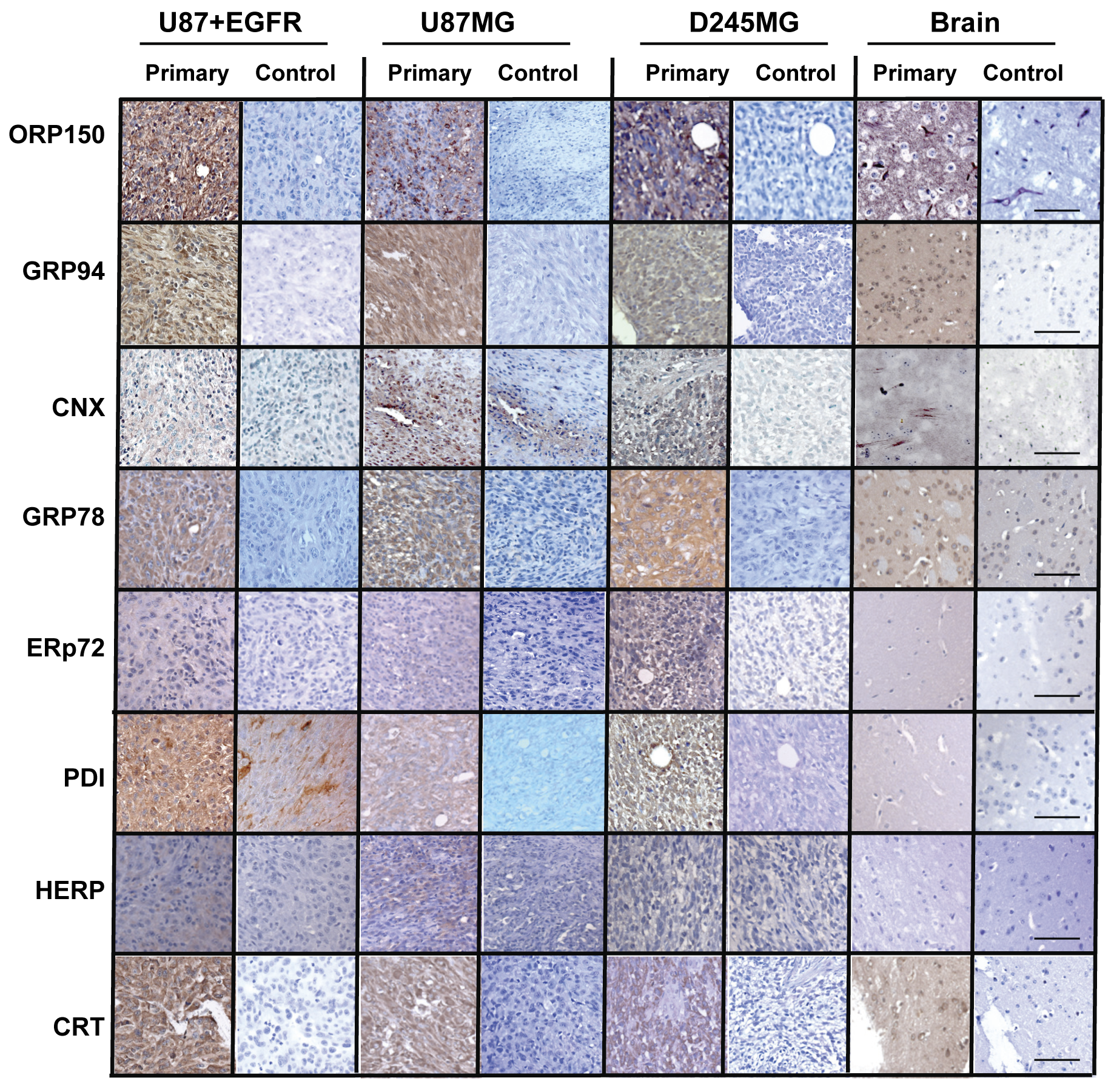

Supplement: Figure S2 — Upregulation of ER resident protein expression in xenograft human gliomas seen in immunohistochemistry. Representative immunohistochemical staining from paraffin-embedded, formaldehyde-fixed tissue sections of normal brain from nu/nu mice and xenografts from U87+EGFR, U8MG7, and D245MG samples. Control panels are probed with a species-matched irrelevant antibody at concentrations identical to the experimental/primary antibody. The scale bar represents 100 µm. (TIF) [file pone.0073267.s002.tif]

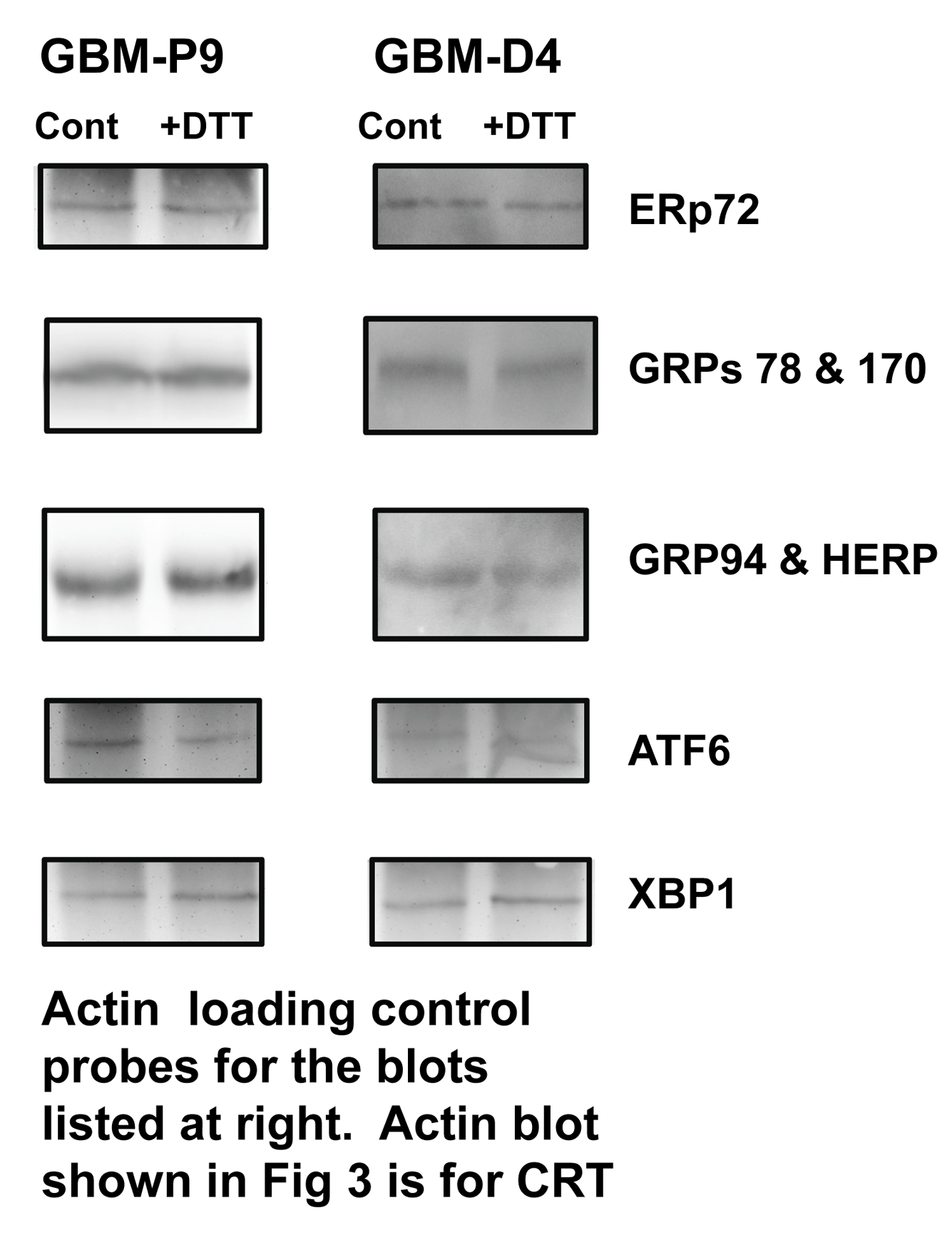

Supplement: Figure S3 — Replicate or stripped/reprobed blots from Figure 3 probed with an anti-actin antibody as a loading control. Blots for GRPs 170 and 78, for ERp72, and for CRT were stripped and re-probed with an actin antibody. Blots for GRP94 and HERP, for ATF6, and for XBP-1 are replicate blots. (TIF) [file pone.0073267.s003.tif]

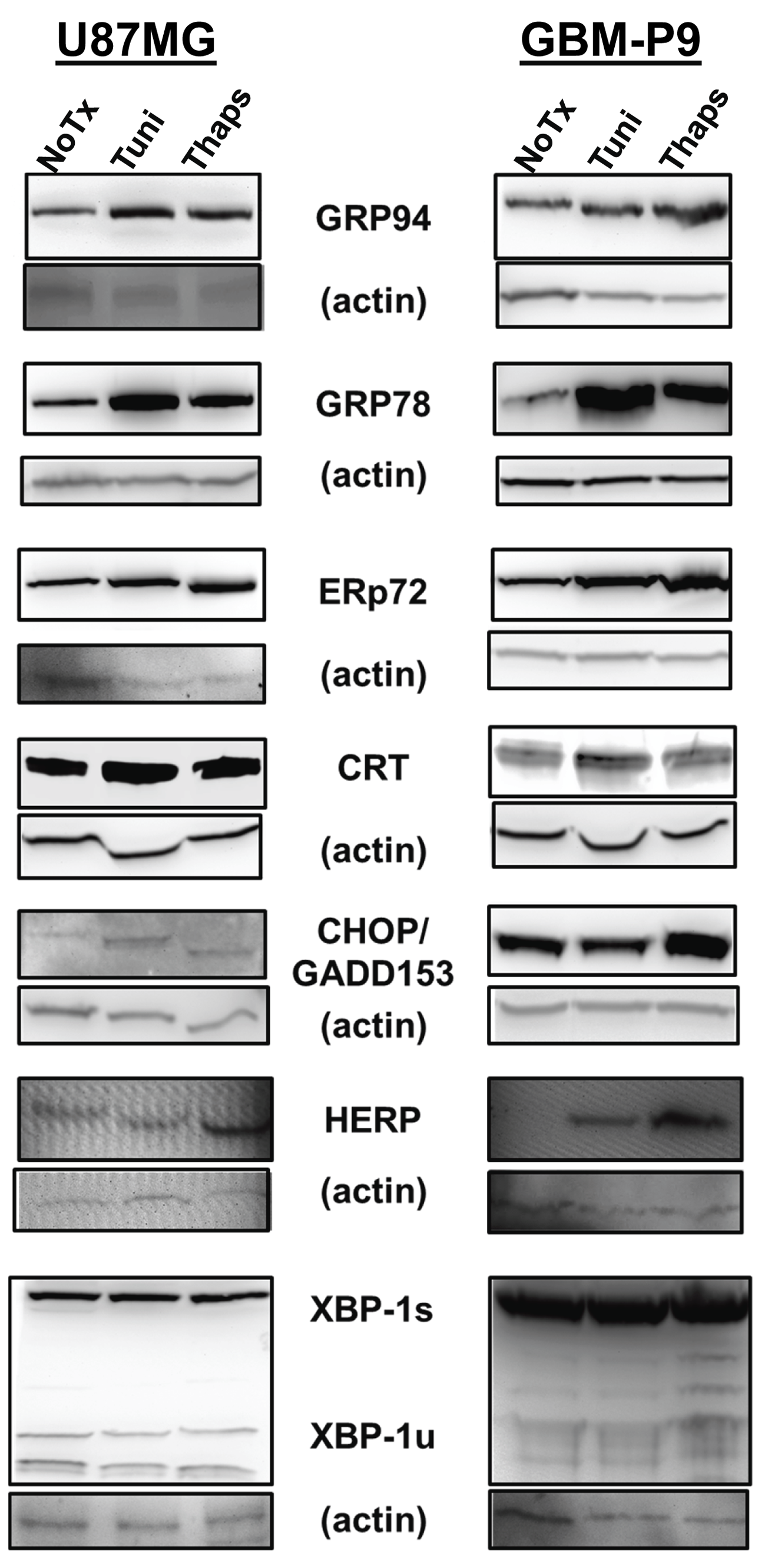

Supplement: Figure S4 — Treatment of glioma cell cultures with other chemical inducers upregulates UPR-related protein expression. U87MG cells and the primary GBM culture model GBM-P9 were treated left untreated (“NoTx”) or were treated with tunicamycin (“Tuni”) or thapsigargin (“Thaps”) as described in Figure 4. Cells were harvested, lysed, and proteins separated by SDS-PAGE, followed by transfer to nitrocellulose for probing in Western blots with the antibodies listed (and their respective actin loading controls). Blotsfor GRP94, GRP78, and ERp72 were stripped and re-probed with actin antibodies. Blots forCRT, CHOP, HERP, and XBP-1 are replicates probed with actin antibodies. (TIF) [file pone.0073267.s004.tif]

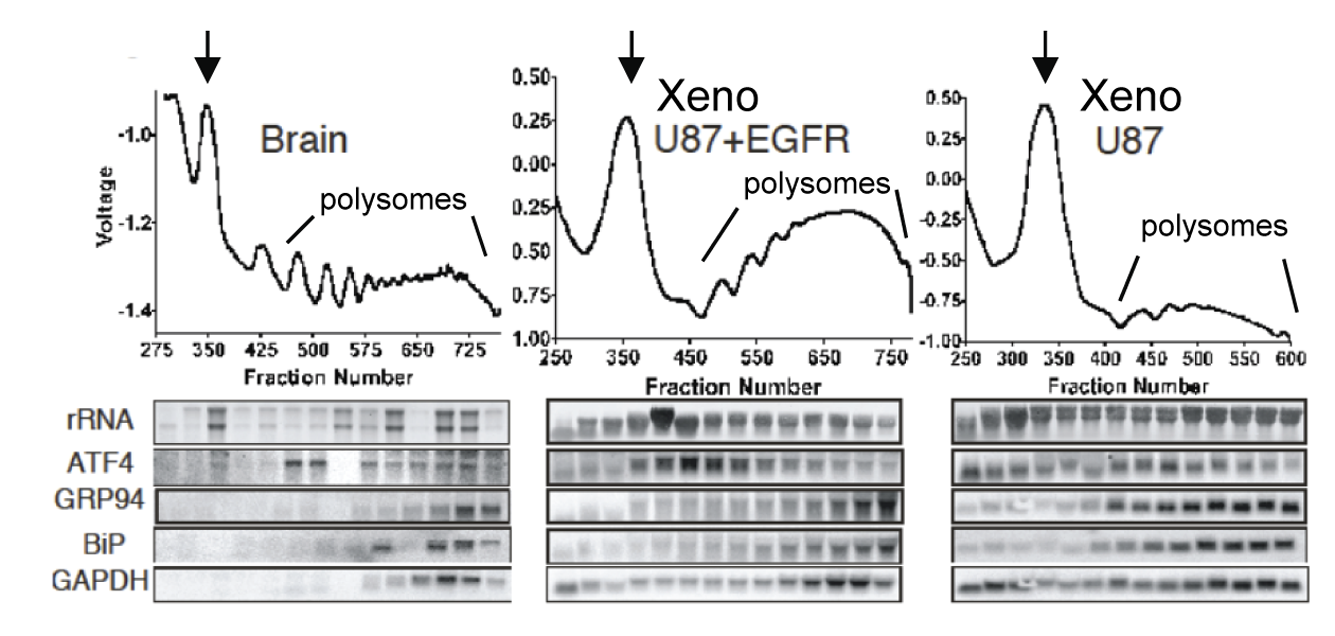

Supplement: Figure S5 — Xenograft tumors exhibit steady-state polyribosome loading of UPR-response transcripts. Polyribosomes were obtained from normal murine brain and solid tumors of both the U87MG and U87+EGFR glioma models. Following homogenization, sample lysates were layered over a linear sucrose gradient (15-50%), separated at 150,000x g for 3 hours, and the gradients fractionated with an automated gradient fractionator, with continuous UV (254 nm) absorbance monitoring. Downward-pointing arrows indicate sedimentation of 80S monosomes. RNA was extracted from individual gradient fractions and analyzed via Northern blot for ATF4, GRP94, BiP/GRP78 and GAPDH mRNA content. (TIF) [file pone.0073267.s005.tif]

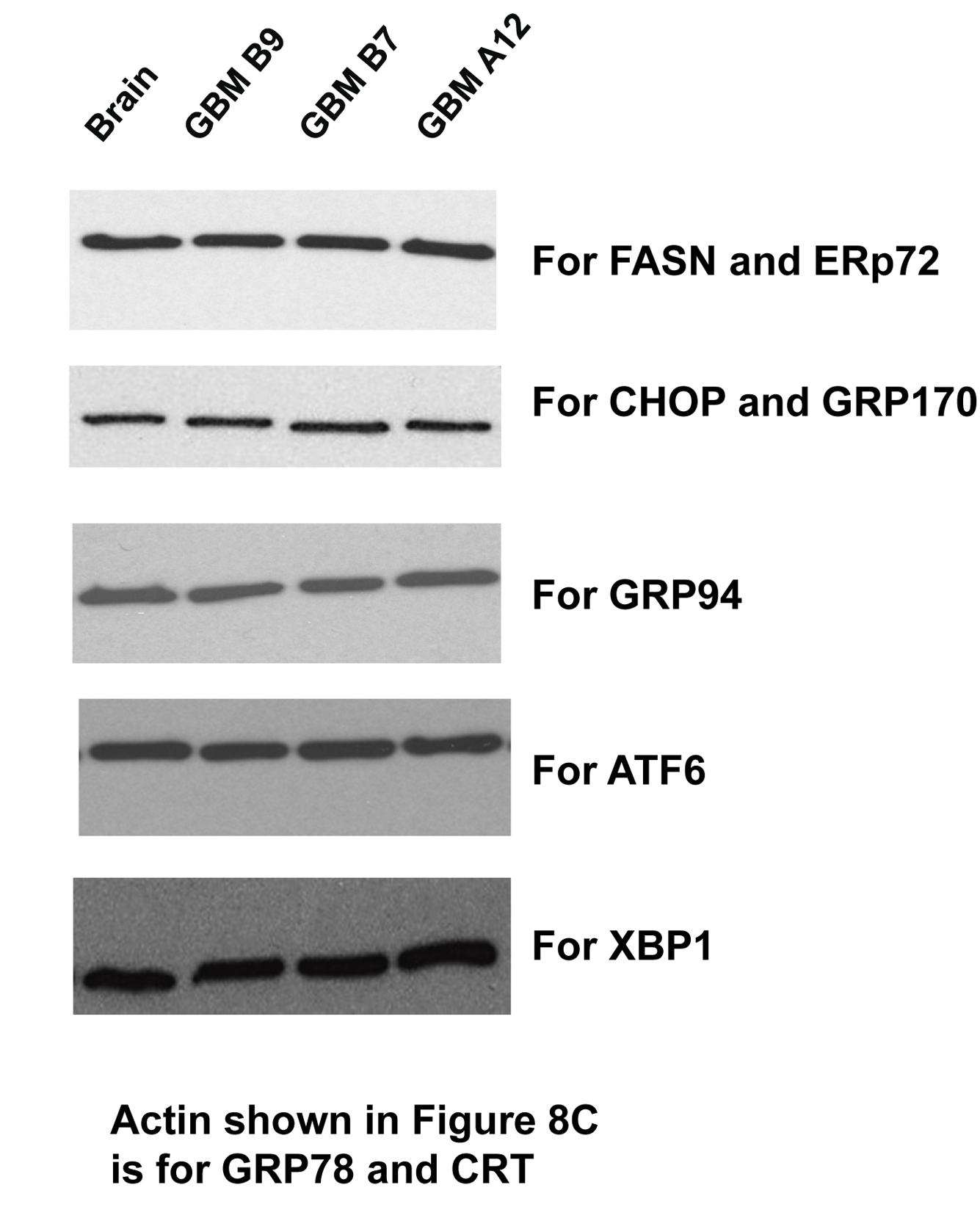

Supplement: Figure S6 — Replicate or stripped/reprobed blots from Figure 8C probed with an anti-actin antibody as a loading control. Blots for FASN and ERp72, for GRP170 and CHOP, for ATF6, for XBP-1, and for GRP78 and CRT, are replicate blots. Blots for GRP94 were stripped and re-probed for actin. (TIF) [file pone.0073267.s006.tif]
